# The Ribosomal Protein Rpl22 Controls Ribosome Composition by Directly Repressing Expression of Its Own Paralog, Rpl22l1

**DOI:** 10.1371/journal.pgen.1003708

**Published:** 2013-08-22

**Authors:** Monique N. O'Leary, Katherine H. Schreiber, Yong Zhang, Anne-Cécile E. Duc, Shuyun Rao, J. Scott Hale, Emmeline C. Academia, Shreya R. Shah, John F. Morton, Carly A. Holstein, Dan B. Martin, Matt Kaeberlein, Warren C. Ladiges, Pamela J. Fink, Vivian L. MacKay, David L. Wiest, Brian K. Kennedy

**Affiliations:** 1 Department of Biochemistry, University of Washington, Seattle, Washington, United States of America; 2 Buck Institute for Research on Aging, Novato, California, United States of America; 3 Blood Cell Development and Cancer Keystone, Immune Cell Development and Host Defense Program, Fox Chase Cancer Center, Philadelphia, Pennsylvania, United States of America; 4 Department of Immunology, University of Washington, Seattle, Washington, United States of America; 5 Department of Comparative Medicine, University of Washington, Seattle, Washington, United States of America; 6 Institute for Systems Biology, Seattle, Washington, United States of America; 7 Department of Pathology, University of Washington, Seattle, Washington, United States of America; The University of North Carolina at Chapel Hill, United States of America

## Abstract

Most yeast ribosomal protein genes are duplicated and their characterization has led to hypotheses regarding the existence of specialized ribosomes with different subunit composition or specifically-tailored functions. In yeast, ribosomal protein genes are generally duplicated and evidence has emerged that paralogs might have specific roles. Unlike yeast, most mammalian ribosomal proteins are thought to be encoded by a single gene copy, raising the possibility that heterogenous populations of ribosomes are unique to yeast. Here, we examine the roles of the mammalian *Rpl22*, finding that *Rpl22^−/−^* mice have only subtle phenotypes with no significant translation defects. We find that in the *Rpl22^−/−^* mouse there is a compensatory increase in *Rpl22-like1* (*Rpl22l1*) expression and incorporation into ribosomes. Consistent with the hypothesis that either ribosomal protein can support translation, knockdown of *Rpl22l1* impairs growth of cells lacking *Rpl22*. Mechanistically, Rpl22 regulates *Rpl22l1* directly by binding to an internal hairpin structure and repressing its expression. We propose that ribosome specificity may exist in mammals, providing evidence that one ribosomal protein can influence composition of the ribosome by regulating its own paralog.

## Author Summary

Translation is the process by which proteins are made within a cell. Ribosomes are the main macromolecular complexes involved in this process. Ribosomes are composed of ribosomal RNA and ribosomal proteins. Ribosomal proteins are generally thought to be structural components of the ribosome but recent findings have suggested that they might have a regulatory function as well. A growing number of human diseases have been linked to mutations in genes encoding factors involved in ribosome biogenesis and translation. These include developmental malformations, inherited bone marrow failure syndromes and cancer in a variety of organisms. Here, we describe the role of one ribosomal protein regulating another. We provide evidence that ribosomal proteins can influence the composition of the ribosome, which we hypothesize, may impact the function of the ribosome.

## Introduction

Protein synthesis is a major energy consuming process involving intricate coordination of translation machinery in response to nutrient availability and stress sensing signals, as well as hormonal and growth factor cues in multi-cellular organisms. The ribosome is comprised of two ribonucleoprotein subunits: the 40S and 60S (‘small’ and ‘large’ subunits, respectively). Together these subunits facilitate peptide bond formation, performing different roles during translation. Ribosome synthesis is a highly controlled process, whereby three distinct RNA polymerases are synchronously coordinated to produce equimolar amounts of four rRNAs and 79 mammalian ribosomal proteins (RPs) [1]–[4].

A growing number of human diseases have been linked to mutations in genes encoding factors involved in ribosome biogenesis and protein synthesis [5], [6]. These include developmental malformations, inherited bone marrow failure syndromes and cancer in a variety of organisms [5], [7]–[9]. In addition, interventions leading to reduced translation, such as dietary restriction and reduced 60S ribosomal protein expression, elicits lifespan extension in yeast, worms and files [10]–[13]. Determining the molecular pathology underlying diseases and the role of ribosomes in aging requires a better understanding of ribosome specificity and the functions of individual RPs.

RPs are generally thought to be essential components of the functional ribosome and although they do not play a direct role in catalyzing peptidyl transfer, they may be critical for both regulatory and structural functions of the ribosome [14], [15]. In addition to their role in the ribosome, many RPs, including murine Rpl22, have been shown to have extra-ribosomal functions [16]–[18]. In particular, as RNA binding proteins, RPs have been found to bind cellular and viral RNAs outside of the context of the ribosome. Some RPs also function to regulate their own expression, such as Rpl30 in yeast [19], [20] and human RPS13 [21].

RPs are often essential for viability. For example, embryonic lethality was reported in the first murine knockout of a *ribosomal protein* (*RP*) gene, *Rps19* [22]. Rpl24 [23], [24], and Rps6 [25]–[27], also play essential roles. However, two reports have found that mice lacking either *Rpl22* or *Rpl29* survive without these *RP* genes [28], [29]. In yeast, approximately 85% of the *RP* genes are duplicated as a result of an ancient genome duplication event [30] and many of these paralogous genes are functionally redundant [31]. Generally, deletion of either paralog, but not both simultaneously, results in viability; however, yeast that lack non-duplicated *RP* genes or both paralogs of an individual subunit are often, but not always, inviable [32]. Cross-complementation studies in yeast, analyzing defects in growth, have shown that most *RP* paralogs are functionally redundant [31]; however, several recent studies suggest that some paralogs might have subtle functional differences [33]–[38]. In the case of *RPL22*, however, tetrad analysis indicated that the *rpl22aΔ rpl22bΔ* double mutant was viable, although slow growing [37], [39], while in worms disruption of *rpl22* expression is lethal (http://www.shigen.nig.ac.jp/c.elegans/index.jsp).

Rpl22 is an external protein on the 60S ribosomal subunit that is incorporated into the ribosome at later stages of ribosome maturation [40]. An early study suggested that Rpl22 was not required for translation *in vitro* [41]; however, the protein has been identified as a component of the ribosome [40] and likely plays a role in protein translation. In addition, other activities have been attributed to Rpl22 in mammals, including association with both viral RNAs, like EBER1, and cellular RNAs, such as human telomerase RNA [42]–[44]. Mice lacking *Rpl22* are viable but have a defect in T cell development attributed to p53-dependent arrest of the αβ lineage T cells [28]. Recently, *RPL22* has been found to be mutated or downregulated in various cancers, including T-acute lymphoblastic leukemias [45], invasive breast carcinoma [46], and lung adenocarcinoma [47].

Here we report evidence that in mice one ribosomal protein can control composition of the ribosome by regulating expression of its own paralog. Knocking out *Rpl22* results in up-regulation of *Rpl22-like1* (*Rpl22l1*), a paralog of *Rpl22* whose predicted protein sequence is highly homologous to Rpl22. *Rpl22l1* was first identified (although mis-labeled Rpl22) in a screen for 14-3-3ε binding partners in mouse brain [48] and has been identified as a trace component of ribosomes in mouse liver and mammary gland tissues [49]. We find that Rpl22l1 co-sediments with actively translating ribosomes in *Rpl22^−/−^* mice and a compensatory increase in Rpl22l1 expression likely accounts for the lack of translational defects in these animals. Enhanced *Rpl22l1* expression also occurs upon acute knockdown of *Rpl22* expression, indicating that Rpl22 has an active role in suppressing the synthesis of its paralog. Mechanistically, we find that Rpl22 directly represses expression of *Rpl22l1* mRNA by binding to an internal hairpin structure. shRNA-mediated knockdown of Rpl22l1 causes a severe growth defect in cells lacking *Rpl22*. Accordingly, we demonstrate that the composition of the ribosome is regulated by the novel mechanism of direct repression of one paralog by another, and offer the hypothesis that this is one mechanism by which ribosome specificity is coordinated.

## Results

### *Rpl22^−/−^* mice are viable

A gene-trapped mouse embryonic stem cell clone harboring a mutation in *Rpl22* was obtained from Bay Genomics and used to generate *Rpl22* heterozygous mice (*Rpl22^+/−^*) (**see Text S1**). 5' rapid amplification of cDNA ends (5′ RACE) followed by automated DNA sequencing determined that the gene-trap vector inserted between the third and fourth exons of *Rpl22* (**Figure S1A**). Gene-trap vector disruption of *Rpl22* expression was confirmed by PCR and western blot analysis (**Figure S1B**). Mice heterozygous for the *Rpl22* mutation (*Rpl22^+/−^*) were interbred to obtain homozygous *Rpl22*-null (*Rpl22^−/−^*) mice. Surprisingly, Mendelian ratios of *Rpl22^+/+^*, *Rpl22^+/−^*, *and Rpl22^−/−^* were found in the resulting progeny. During the construction of our mouse line, Anderson et al. reported the generation of viable *Rpl22^−/−^* mice and observed defects in lymphocyte development [28].

Characterization of our *Rpl22^−/−^* mice indicated that they have defects in lymphocyte development (**Figure S2, S3, S4**) similar to that described by Anderson et al. (2007) [28]. B220+ B cells in the bone marrow were also significantly reduced in *Rpl22^−/−^* mice (**Figure S4A, B**). Further analysis indicated that B cell development was interrupted by *Rpl22* deficiency, as evidenced by a decrease in the B220+ developing IgM-IgD- and immature IgM+IgD- B cells (**Figure S4C, D**).

Despite the ubiquitous expression of Rpl22 and its hypothesized role in mRNA translation, disruption of *Rpl22* in mice results in a remarkably mild phenotype. Hematologic parameters are normal in these mice [50]. Also, unlike deletion of *RPL22* in yeast [51], *Rpl22^−/−^* mice have no substantial difference in growth rate or size relative to *Rpl22^+/+^* and *Rpl22^+/−^* littermates (Anderson et al. 2007 and our unpublished data). Surprisingly, no significant differences were observed in the polysome profiles of lysate from *Rpl22^−/−^* liver, lung or cultured ear fibroblasts when compared to samples collected from *Rpl22^+/+^* mice (unpublished data), indicating that Rpl22 is not essential for translation efficiency or ribosome biogenesis in the tissues evaluated.

### Compensation by Rpl22l1 in Rpl22-null mice

**Figure 1:**
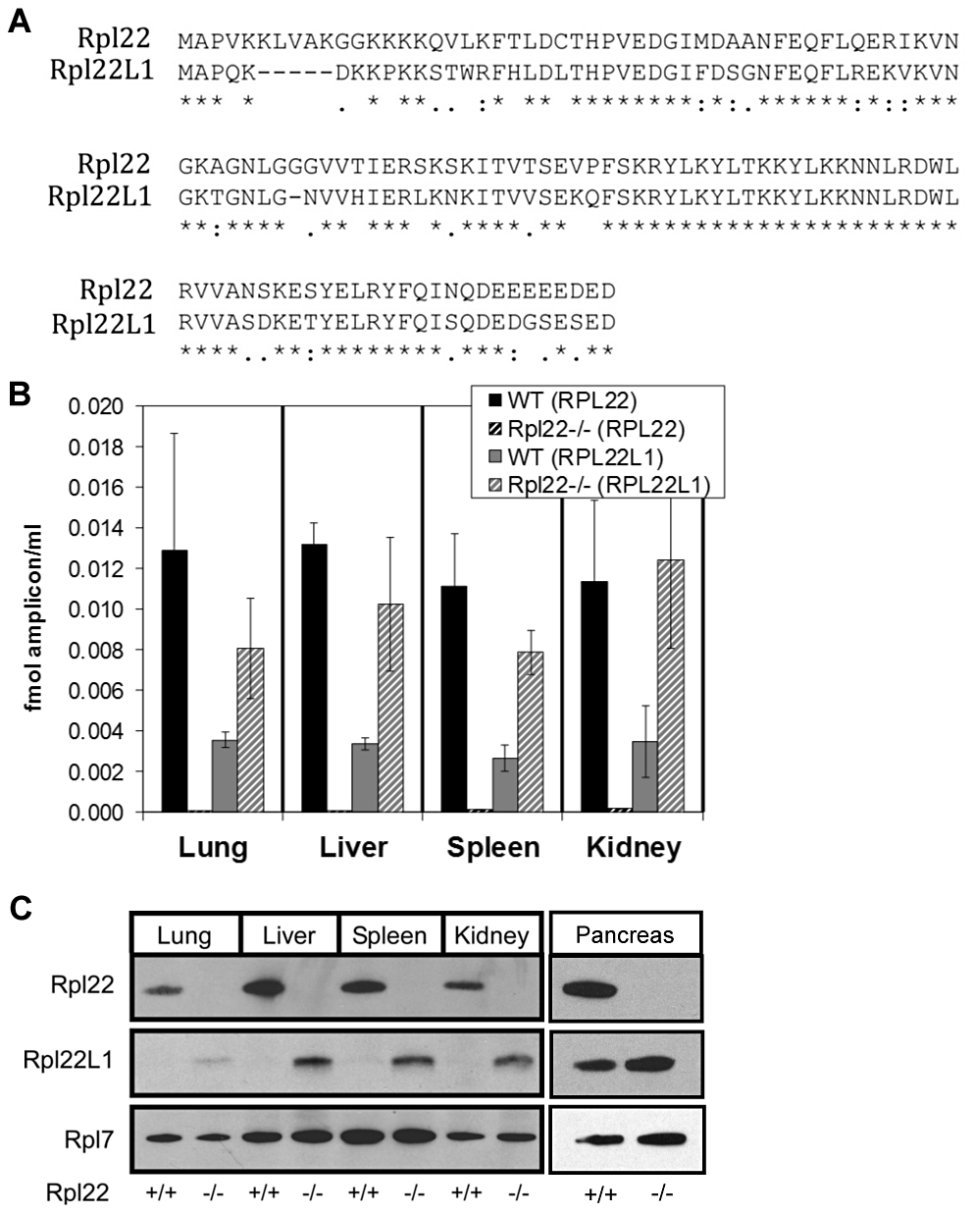
Mouse Rpl22 has an expressed paralog, Rpl22l1. (**A**) Alignment of Rpl22 and Rpl22l1 protein sequences. Lung, liver, spleen, kidney and pancreas, harvested from *Rpl22^−/−^* mice and their littermate controls, were analyzed for relative *Rpl22* and *Rpl22l1* mRNA levels by qRT-PCR (**B**) or protein expression by Western blot analysis (**C**). Results are representative of 3 independent experiments.

We considered the possibility that another factor might be compensating for lack of Rpl22 in mice. A bioinformatic search identified Rpl22-like1 (Rpl22l1) as a candidate. *Rpl22l1* encodes a 122 amino acid protein that is 73% identical to Rpl22 (**Figure 1A**). The protein sequence of Rpl22l1 is highly conserved from human to zebrafish (**Figure S5**). To determine if significant levels of *Rpl22l1* mRNA exists in tissues from *Rpl22^+/+^* mice and whether *Rpl22l1* transcript levels increase in *Rpl22^−/−^* mice, lung, liver, spleen and kidney were harvested from *Rpl22^−/−^* mice and their littermate controls and analyzed by quantitative RT-PCR (qRT-PCR) for *Rpl22* and *Rpl22l1* expression with Acidic Ribosomal Protein (ARBP) mRNA levels used for normalization (**Figure 1B**). In *Rpl22^+/+^* samples, high *Rpl22* expression was detected, while *Rpl22l1* transcripts were less abundant. In samples isolated from *Rpl22^−/−^* mice, qRT-PCR revealed a ∼3-fold induction of *Rpl22l1* mRNA expression relative to littermate controls.

*Rpl22l1* transcripts were found associated with actively translating ribosomes in both *Rpl22^+/+^* and *Rpl22^−/−^* mouse ear fibroblast samples (unpublished data), suggesting that the *Rpl22l1* mRNA is actively translated into protein. Consistently, equivalent increases in Rpl22l1 protein levels are observed in the absence of *Rpl22* in lung, liver, spleen and kidney (**Figure 1C**). Similar increases in *Rpl22l1* mRNA and protein expression were observed in *Rpl22^−/−^* skeletal muscle and brain (unpublished data). Additionally, Rpl22l1 was found to be abundantly expressed in both *Rpl22^+/+^* and *Rpl22* ^−/−^ pancreas (**Figure 1C**). These results indicate that Rpl22 negatively regulates, either directly or indirectly, *Rpl22l1* expression in a range of mouse tissues.

### Rpl22l1 protein co-sediments with actively translating ribosomes

**Figure 2:**
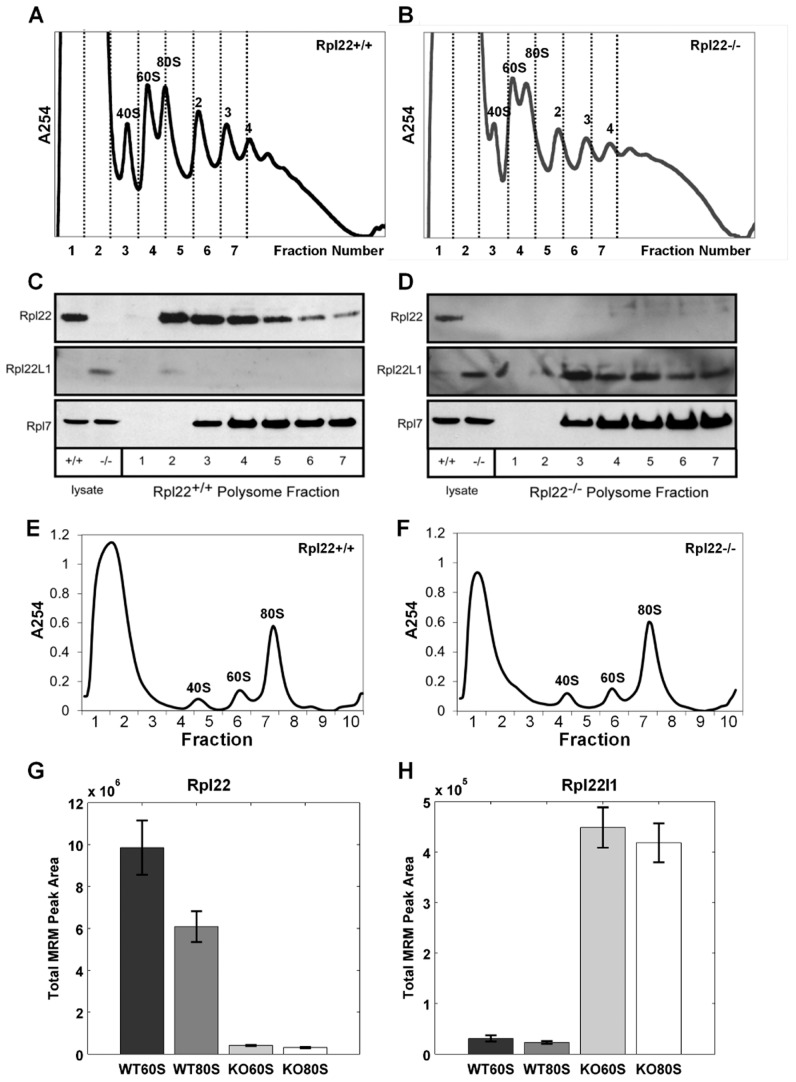
Both mouse Rpl22 and Rpl22l1 proteins can be incorporated into ribosomes. Liver tissue was isolated from *Rpl22^+/+^* (**A, C**) and *Rpl22^−/−^* (**B, D**) mice then, after sedimentation of the lysates on sucrose gradients, fractions were collected and loaded onto an SDS-page gel for western blot analysis (**C** and **D**, respectively). Images are representative of 3 independent experiments. Multiple Reaction Monitoring Mass spectrometry (MRM-MS) analysis was performed on free 60S subunits and 80S monosomes from actively translating polysomes. Liver lysates from *Rpl22^+/+^* and *Rpl22^−/−^* mice were subjected to a brief treatment with low amounts of RNase A to degrade mRNA between ribosomes in polysomes and release the ribosomes as 80S monomers. After inhibiting the RNase with KCl and heparin, the samples were fractionated on 10–30% sucrose gradients containing 800 mM KCl to disrupt any “nonproductive couples” of 40S and 60S subunits [79]. (**E, F**) Representative gradient profiles for Rpl22+/+ and Rpl22−/−samples, respectively. Fraction 6 was used to isolate 60S subunits for MRM-MS, while fraction 7 was used to isolate 80S monosomes. Summation of the integrated MRM peak areas for all transitions from all observed peptides for Rpl22 (**G**) and Rpl22l1 (**H**) proteins yielded the total MRM peak areas plotted for each of the four tested samples (WT60S, WT80S, KO60S, KO80S), indicating the relative amounts of Rpl22 and Rpl22l1 in these samples. The height of each bar represents the average of the three technical replicates performed for the given sample, and each error bar represents +/−1 standard deviation. p-values>3E-3 in both cases by paired student's t-test. Rpl22 peptides: AGNLGGGVVTIER; ITVTSEVPFSK; YFQINQDEEEEEDED. Rpl22l1 peptides: TGNLGNVVHIER; ITVVSEK.

To determine if Rpl22l1 is incorporated into actively translating ribosomes, liver tissue was isolated from *Rpl22^−/−^* mice and their littermate controls followed by sedimentation of the lysates on sucrose gradients. Fractions collected from the gradients, were subsequently loaded onto an SDS-page gel for western blot analysis. In *Rpl22^−/−^* samples, Rpl22l1 is present in fractions containing 60S ribosome subunits and polysomes, suggesting that it is incorporated into free ribosome subunits and ribosomes actively translating mRNA in the absence of *Rpl22* **(** **Figure 2** **)**. Rpl7, a RP that is incorporated into the large subunit of the ribosome, is present in the fractions containing 60S ribosome subunits and polysomes in both *Rpl22^+/+^* and *Rpl22* ^−/−^ samples. Rpl22 and Rpl22l1, but not Rpl7, were detected in fractions 1 and 2, representative of the free, non-ribosomal lysate **(** **Figure 2C, D** **)**, consistent with the hypothesis that these RPs exist in states within the cell both associated with the ribosome and independent of the ribosome. Additionally, while Rpl22l1 levels are relatively evenly detected in 60S containing fractions of the polysome profile **(** **Figure 2D** **, fractions 3–7)** Rpl22 is detected at higher levels in the fractions containing free 60S subunits and messages loaded with fewer ribosomes **(** **Figure 2C** **, fractions 3–5 vs 6–7)**.

To verify that Rpl22l1 was incorporated into 60S subunits and actively translating ribosomes, free 60S subunits and 80S monosomes from actively translating polysomes were isolated from liver lysates of *Rpl22^+/+^* (WT) and *Rpl22^−/−^* (KO) mice using sucrose density gradient fractionation **(** **Figure 2E, F** **; see Text S1)**. Samples were then concentrated and prepared for mass spectrometry analysis using standard methods **(see Text S1)**. In order to measure the relative amounts of Rpl22 and Rpl22l1 in the WT and KO mouse liver lysate samples, we used multiple reaction monitoring (MRM), a targeted mass spectrometry (MS) approach that is highly sensitive. MRM is a targeted type of MS and requires a list of peptide targets and their subsequent fragment targets—known as a transition list—to program the analysis on the instrument **(see Text S1)**. The final MRM analysis consisted of 4 peptide targets for Rpl22 (5 counting uniquely modified targets) and 3 peptide targets for Rpl22l1, and was limited to the top 8 fragment ions per peptide, creating a total of 64 transitions targeted in the analysis.

Summing the integrated MRM peak areas for all transitions from all observed peptides for each protein (Rpl22 and Rpl22l1) yielded the total MRM peak areas plotted for each of the four tested samples (WT60S, WT80S, KO60S, KO80S) and indicated the relative amounts of Rpl22 and Rpl22l1 in these samples (**Figure 2G, H**). These data indicate that Rpl22l1 levels are significantly higher in the 60S and 80S subunits isolated from *Rpl22^−/−^* liver than in *Rpl22^+/+^* littermate controls (**Figure 2H**), supporting the hypothesis that Rpl22 regulates Rpl22l1 expression and, as a result, incorporation into ribosomes.

### Rpl22 directly regulates *Rpl22l1* expression

**Figure 3:**
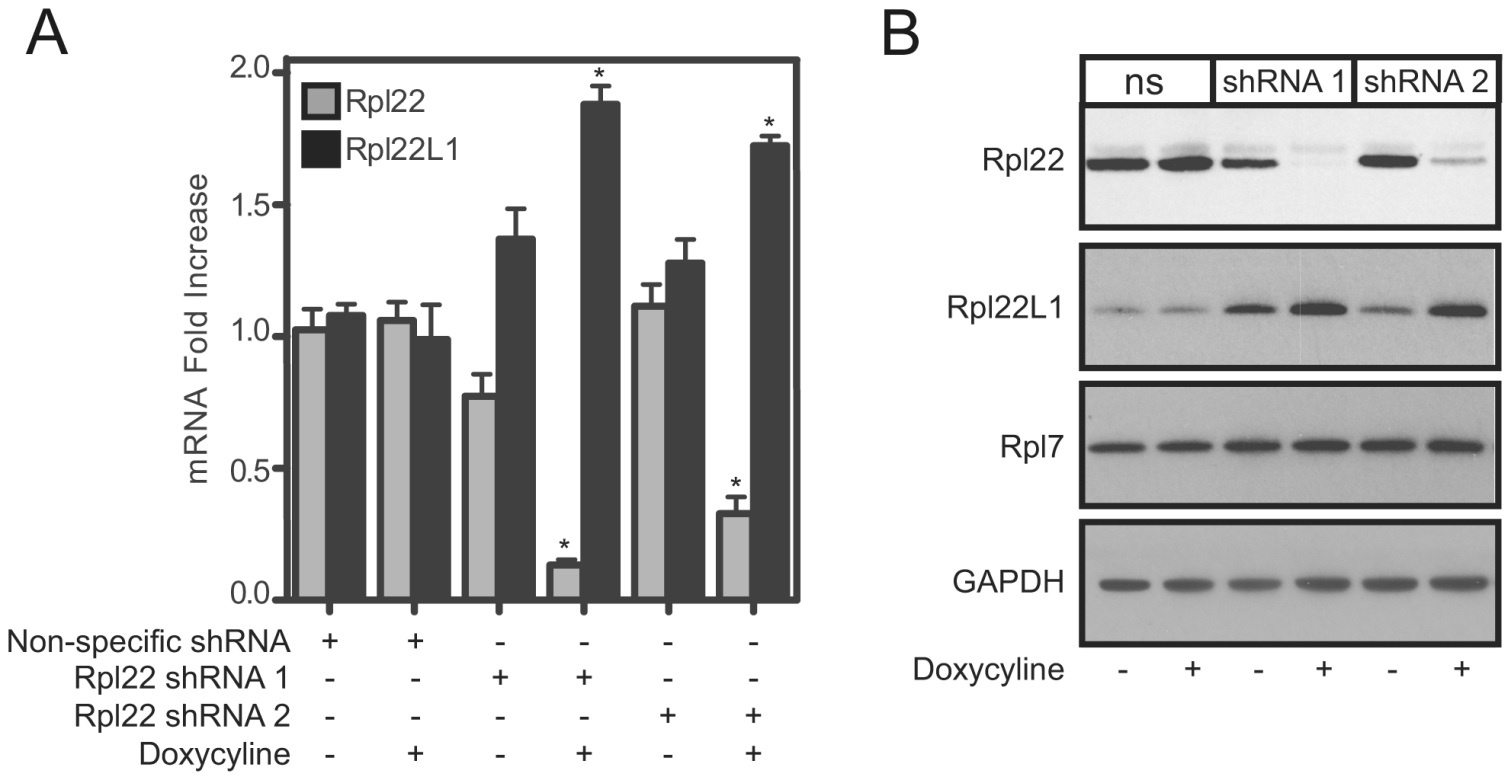
Acute knockdown of Rpl22 enhances Rpl22l1 expression. 3T9 cells were transduced with doxycycline-inducible shRNA lentiviral constructs directed at *Rpl22* (shRNA 1 and shRNA 2) or a non-specific (ns) shRNA construct and treated with doxycycline for 3 days to induce shRNA expression. NS, shRNA 1, or shRNA 2 expressing cells were analyzed for relative *Rpl22* and *Rpl22l1* mRNA levels by qRT-PCR (**A**) or protein expression by Western blot analysis (**B**). Results are the average ± SEM of 4 independent experiments (**A**) or representative of 3 independent experiments (**B**). Statistical significance is indicated (*; p<0.001 compared to untreated control).

Why does expression of *Rpl22l1* increase in mouse tissues lacking *Rpl22*? We considered two potential explanations: (1) Rpl22 directly regulates *Rpl22l1* expression, or (2) compensation occurs during development in *Rpl22^−/−^* mice. To distinguish between these two possibilities, *Rpl22* was acutely knocked down in 3T9 fibroblasts using a lentiviral-mediated inducible knockdown system that allows doxycycline-inducible regulation of *Rpl22* and changes in Rpl22l1 expression were examined. 3T9 cells were transduced with 2 different tet-on shRNA lentivirus constructs (shRNA 1 and shRNA 2) that target *Rpl22* mRNA or a nonspecific control construct. Following 3 days of doxycycline treatment, *Rpl22l1* mRNA expression is enhanced 1.8 fold in 3T9 cells with reduced *Rpl22* expression (**Figure 3A**). Western blot analysis confirmed that Rpl22l1 protein levels were elevated by the knockdown of *Rpl22*, while expression of other RPs, such as Rpl7 remained unchanged (**Figure 3B**). These results confirm that Rpl22 negatively regulates the expression of Rpl22l1 acutely and raise the possibility that Rpl22-mediated regulation of Rpl22l1 is an active process with biological significance.

**Figure 4:**
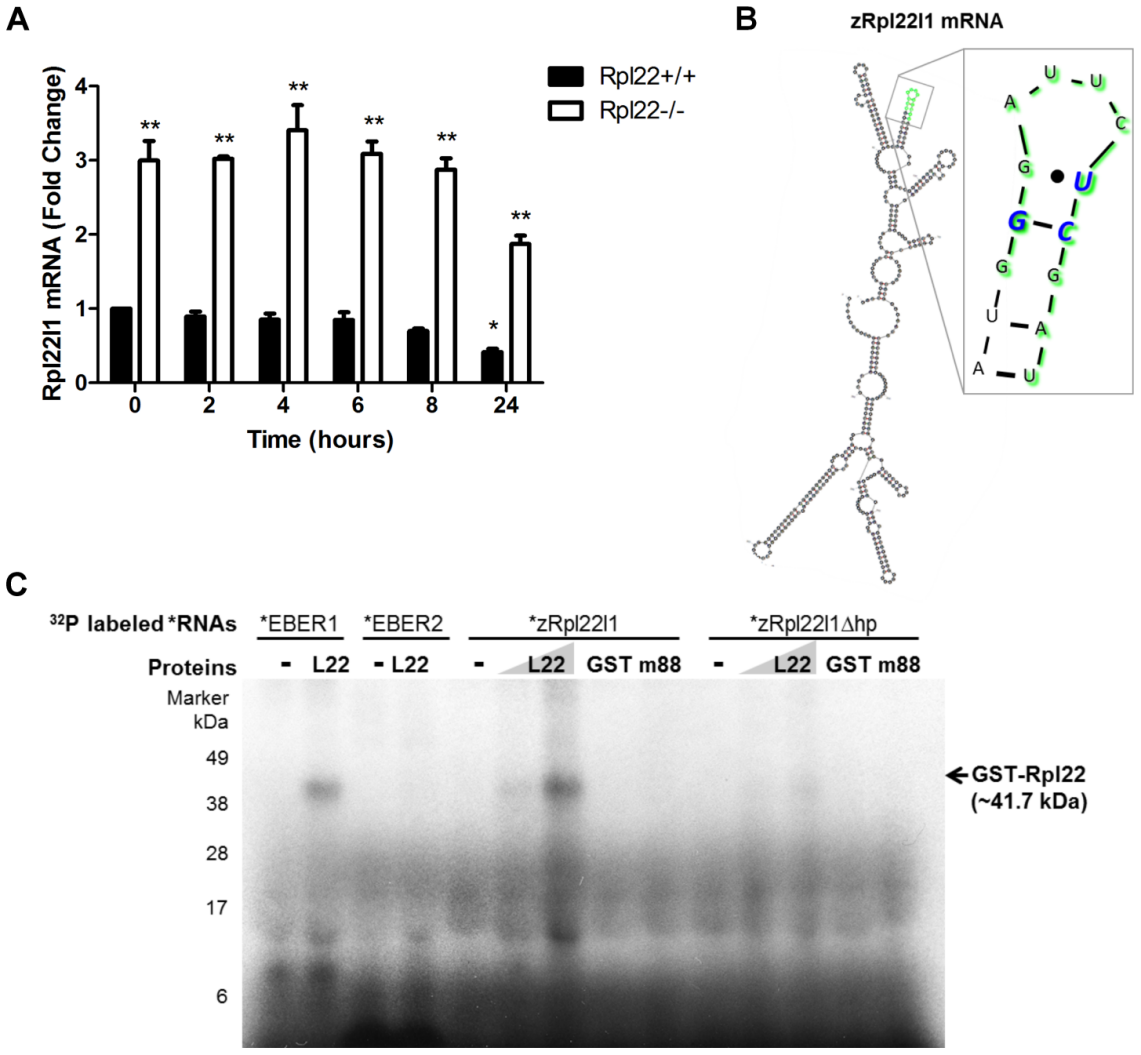
Rpl22 directly binds *Rpl22l1* mRNA to regulate its expression levels. (**A**) In the absence of Rpl22, *Rpl22l1* mRNA levels are more stable in the presence of Actinomycin D. Rpl22+/+ or Rpl22−/− 3T9 cells were treated with Actinomycin D (1 µM final concentration) and total RNA was harvested at the time points shown. Levels of *Rpl22l1* mRNA were quantitated by qRT-PCR. Results are the average ± SEM of 3 independent experiments and the statistical significance indicated is (*, p<0.01, compared to *Rpl22+/+* untreated; ** p<0.001, compared to *Rpl22+/+* at each time point). (**B**) M-fold analysis [54] of *zRpl22l1* mRNA reveals the presence of a consensus Rpl22 RNA-binding motif. In green are the residues deleted to remove the hairpin (*zRpl22l1Δhp*). In blue are the residues known to be essential for Rpl22 binding. (**C**) Autoradiogram of ribonuclease protection assay reveals Rpl22 protein binds to *Rpl22l1* mRNA and this binding is abrogated upon removal of the hairpin. 32P labeled *EBER1* (positive control), *EBER 2* (negative control), *zRpl22l1* or *zRpl22l1Δhp* RNAs were incubated in the absence or presence of GST-Rpl22 (41.7 kDa), GST (27 kDa) or m88, a GST-Rpl22 RNA binding mutant (41.6 kDa), as indicated, then UV-cross-linked, digested with RNase A, and run on a SDS protein gel. GST-Rpl22 was detected, hence, bound to *EBER1* and *zRpl22l1* RNAs but not *Rpl22l1Δhp* RNA, indicating Rpl22 binds to *Rpl22l1* mRNA and this binding is abrogated upon removal of the hairpin. Numbers indicate molecular weight protein ladder in kDa.

Collectively, these data suggest that Rpl22 is regulating expression of Rpl22l1 but the mechanism leading to the increased expression is unknown. To determine whether Rpl22 affects the stability of *Rpl22l1* mRNA, cultures of 3T9 cells were treated with actinomycin D, which blocks transcription by all three eukaryotic polymerases [52]. After actinomycin D treatment, the levels of *Rpl22l1* mRNA in Rpl22+/+ 3T9 cells decreased significantly (p<0.01) relative to the untreated control, while in Rpl22−/− 3T9 cells Rpl22l1 levels were maintained and the rate of decay was reduced (**Figure 4A**). These results suggest that Rpl22 affects the stability of *Rpl22l1* mRNA.

If Rpl22 is involved in mediating *Rpl22l1* stability, does Rpl22 bind directly to *Rpl22l1* mRNA? Previous studies determined that Rpl22 is associated with Epstein-Barr virus-expressed RNA, EBER1 [43], [53] and evaluation of the RNA binding specificity of Rpl22 suggested that Rpl22 recognizes a stem loop (hairpin) structure with a G-C at the neck followed by a U [42], [53]. To address whether regulation of Rpl22l1 expression is directly mediated by Rpl22, an algorithm termed M-fold that predicts RNA secondary structure was used to evaluate *Rpl22l1* mRNA structure for potential Rpl22 RNA binding motifs [54]. Analysis revealed the presence of a consensus Rpl22 RNA-binding motif within exon 2 of z*Rpl22l1*, suggesting that Rpl22 might interact directly with *Rpl22l1* mRNA (**Figure 4B**).

To test whether Rpl22 can directly bind *Rpl22l1* mRNA via the hairpin structure identified in *Rpl22L1* mRNA by M-fold analysis an RNAse protection analysis was performed. Recombinant proteins were incubated with radiolabeled RNA, and UV-crosslinked. The RNAs were then digested with RNase A, and samples were run on a protein gel. Proteins bound to radiolabeled RNA were detected by autoradiogram at their expected molecular weight. Unbound proteins are not detected on autoradiogram. Recombinant Rpl22 was found to bind to *in vitro* transcribed *zRpl22L1* mRNA, but not the *zRpl22l1* mRNA lacking the hairpin structure (*zRpl22L1Δhp*) (**Figure 4C**), suggesting the Rpl22 directly binds to *Rpl22l1* mRNA.

**Figure 5:**
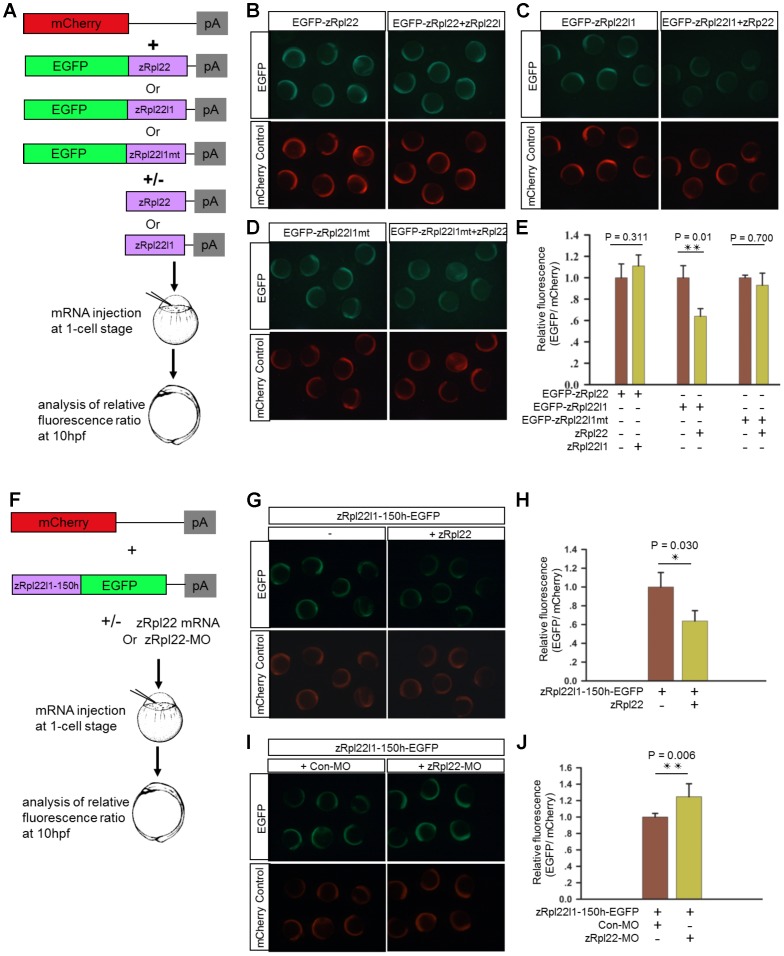
Regulation of *Rpl22l1* mRNA expression is mediated by a hairpin structure. (**A**) Schematic representation of the biosensor quantification assay. (**B–D**) Stereoimages of zebrafish embryos illustrate that co-injection of zRpl22 repressed fluorescence derived from an EGFP-Rpl22l1 fusion protein upon injecting mRNA for both and assessing fluorescence at 6 hours post fertilization. Rpl22, Rpl22l1 or mutated Rpl22l1 (Rpl22l1mt) coding sequence was fused to *EGFP* mRNA and co-injected with *mCherry* mRNA (injection control) along with the corresponding inhibitor mRNAs (*zRpl22* or *zRpl22l1*) into 1-cell stage zebrafish embryos. (**F**) Schematic representation of the experimental procedure. A *zRpl22l1-150h-EGFP* heterologous reporter mRNA, containing the minimal sequence identified by mFOLD to form the hairpin structure, was co-injected with *mCherry* mRNA (injection control) and (**G**) *Rpl22* mRNA or (**I**) Rpl22-Morpholino (Rpl22-MO) into 1-cell stage zebrafish embryos. (**E, H, J**) At 10 hpf, the relative fluorescence intensity was calculated and normalized to control injections (n = 3, each group). Data are shown as mean ± standard deviation (s.d.).

To determine if Rpl22 directly regulates expression of Rpl22l1, we employed a biosensor quantification assay using GFP as a fluorescent indicator of effects on expression. Zebrafish embryos were microinjected with mRNAs *EGFP-zRpl22*, *EGFP-Rpl22l1* or a mutant form of *Rpl22l1* in which the *Rpl22l1* hairpin was modified (*EGFP-zRpl22L1mt*) in combination with constructs expressing *zRpl22* or *zRpl22l1*. *mCherry* mRNA was co-injected to allow for quantification of the relative fluorescence intensity. Co-injection of *zRpl22* with *EGFP-Rpl22l1* led to a significant decreased fluorescence relative to those embryos co-injected with *zRpl22* and *EGFP-Rpl22l1mt* (**Figure 5A–E**), suggesting that the hairpin structure within *Rpl22l1* mRNA is necessary for Rpl22 to directly regulate its expression. Next, to assess if the presence of the hairpin structure within the *Rpl22l1* mRNA is sufficient to regulate expression, the hairpin sequence from *zRpl22l1* mRNA was fused to EGFP and evaluated in the biosensor quantification assay. The heterologous reporter mRNA, *zRpl22l1-150h-EGFP*, containing the minimal sequence identified by M-fold to form the hairpin structure, was co-injected with *mCherry* mRNA (injection control) and **(** **Figure 5G** **)** z*Rpl22* mRNA or **(** **Figure 5I** **)** Rpl22-Morpholino (Rpl22-MO) into zebrafish embryos. Rpl22 repressed the expression of *zRpl22l1-150h-EGFP* reporter, while knockdown Rpl22 can increase the expression of reporter, suggesting the hairpin structure identified in z*Rpl22l1* mRNA is sufficient to regulate mRNA abundance.

### Rpl22l1 incorporation into ribosomes is associated with increased cell proliferation

**Figure 6:**
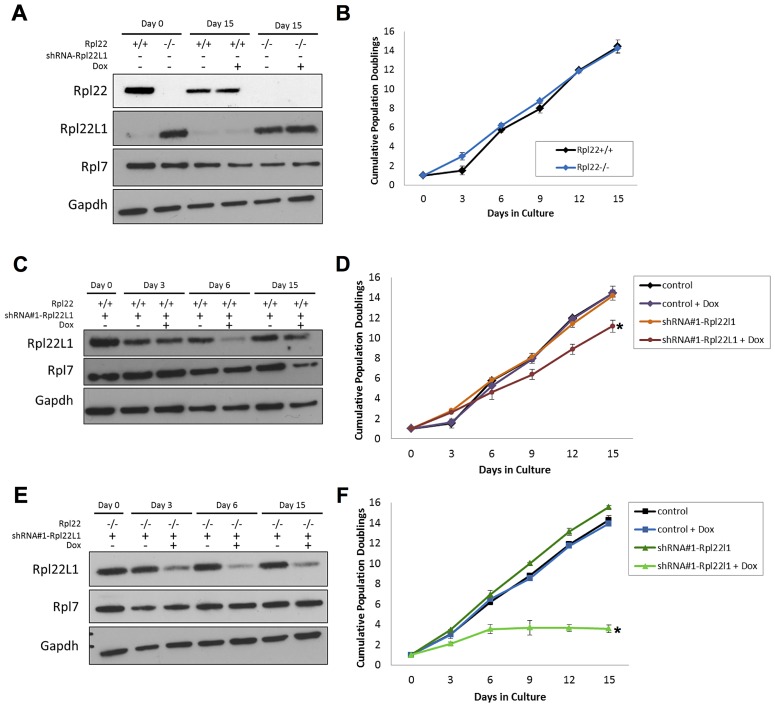
Acute knockdown of Rpl22l1 expression impairs cellular growth. (**A**) Lysates isolated *Rpl22^+/+^* and *Rpl22^−/−^* 3T9 cells treated with or without doxycycline were collected to assess levels of Rpl22 or Rpl22L1 by Western blot analysis. GAPDH was used as a loading control. (**B**) Growth of *Rpl22^+/+^* and *Rpl22^−/−^* 3T9 cells was compared. Levels of Rpl22l1 were analyzed by Western blot analysis to confirm that the Rpl22l1-shRNA knocked down levels of Rpl22l1 in doxycycline-treated *Rpl22^+/+^* (**C**) and *Rpl22^−/−^* (**E**) 3T9 cells transduced with the shRNA construct. Growth of *Rpl22^+/+^* (**D**) and *Rpl22^−/−^* (**F**) 3T9 cells transduced with each shRNA construct was determined. Results are representative of 2 independent experiments with error bars representative of ±SD. Statistical significance is indicated (*, p<0.05 compared to untreated control).

In yeast, ribosomal protein paralogs are thought to functionally compensate for one another, each incorporating into the ribosome in the absence of the other. Deletion of both yeast *RPL22* paralogs results in viable, but slow growing cells [37]. Recently *Rpl22^+/−^* or *Rpl22^−/−^* primary mouse embryonic fibroblasts (MEFs) were found to grow faster and display increased transformation potential relative to MEFs isolated from *Rpl22^+/+^* littermates [45] To further evaluate the effect of Rpl22 and Rpl22Ll1 expression on growth rates, *Rpl22^+/+^* or *Rpl22^−/−^* 3T9 fibroblasts were transduced with one of 2 different tet-on shRNA lentivirus constructs (shRNA#1- and shRNA#2-Rpl22l1) that target *Rpl22l1* mRNA to acutely knock-down its expression. Western blot analysis confirmed that Rpl22l1 protein levels were elevated in *Rpl22^−/−^* 3T9 fibroblasts (**Figure 6A**). No significant difference was observed in the growth rates of *Rpl22^+/+^* or *Rpl22^−/−^* 3T9 fibroblasts (**Figure 6B**). In doxycycline-treated *Rpl22^+/+^* or *Rpl22^−/−^* 3T9 fibroblasts expressing the shRNA constructs, Rpl22l1 protein levels were confirmed to be reduced by western blot analysis (**Figure 6C, E**). Knockdown of Rpl22l1 significantly reduced growth rates of *Rpl22^+/+^* fibroblasts and greatly impaired that of *Rpl22^−/−^* 3T9 fibroblasts (**Figure 6D, F** **and S6**), indicating that cells lacking both paralogs have severe growth defects. In contrast, acute knockdown of Rpl22 in *Rpl22^+/+^* 3T9 fibroblasts resulted in no change in the rate of proliferation (**Figure S7**). In summary, these cell culture studies indicate that expression of at least one paralog of Rpl22 is required for normal growth and suggests that Rpl22l1 may also affect cell proliferation by a mechanism independent of Rpl22.

## Discussion

Here we report on phenotypes of mice lacking the large subunit ribosomal protein, *Rpl22*. Surprisingly, *Rpl22^−/−^* mice have no developmental defects, other than previously reported defects in T and B cell development (Anderson et al. 2007 and our data). More generally, *Rpl22^−/−^* mice have no significant defects in translation, as judged by sucrose gradient sedimentation to detect ribosome occupancy on transcripts (unpublished data). This is somewhat surprising since yeast lacking *Rpl22* function are slow growing with substantial defects in translation [10], [37]. One possible explanation is that the lack of a defect in translation in *Rpl22^−/−^* mice is explained by compensatory increases in expression of another gene, *Rpl22l1*, which shares a high degree of homology with *Rpl22*. While *Rpl22l1* mRNA is detected at low levels in most tissues of wild-type mice, mRNA and protein levels are dramatically increased in the *Rpl22* knockout. It is interesting that Rpl22l1 is expressed at relatively high levels in the pancreas even though Rpl22 is present as well. Future studies will be needed to determine why these two paralogs are jointly expressed in this tissue. When expressed, Rpl22l1 was incorporated into ribosomes and actively translating polysomes. These findings indicate that Rpl22l1 is capable of functioning within ribosomes that are actively translating mRNA. Future studies examining the function of Rpl22l1 will help decipher to what extent Rpl22 and Rpl22l1 may have redundant roles and also determine their independent functions, for which accumulating evidence is emerging [28], [45] (O'Leary et al, unpublished data).

Although poorly understood, gene compensation during development is a recurrent phenomenon in mouse knockout studies [55]; therefore, enhanced expression of *Rpl22l1* might reflect developmental compensation in *Rpl22^−/−^* mice. Alternatively, Rpl22 could play an active role in the repression of Rpl22l1. To test this, we examined the consequences of acute knockdown of *Rpl22* in 3T9 fibroblasts. Our findings indicate that *Rpl22l1* mRNA and protein levels are rapidly increased following *Rpl22* knockdown and support the conclusion that developmental compensation does not account for the increased *Rpl22l1* mRNA and protein levels. Instead, we find that inhibition of transcription in cells lacking Rpl22 results a slower decay of *Rpl22l1* mRNA compared to Rpl22+/+ cells, suggesting Rpl22 affects the stability of *Rpl22l1* mRNA. It is possible that more than one mechanism is involved in increasing Rpl22l1 expression in the absence of Rpl22. Further studies interrogating other mechanisms are needed to full understand what regulates Rpl22l1 upon Rpl22 deficiency. We find that Rpl22 binds to a hairpin structure in the *Rpl22l1* mRNA. The hairpin motif identified in *Rpl22l1* mRNA is necessary and sufficient for regulation of mRNA abundance by Rpl22. These data suggest that Rpl22 might function in an extra-ribosomal capacity to bind and destabilize *Rpl22l1* mRNA. *Rpl22l1* is yet another RNA demonstrated to interact with Rpl22. The viral RNA *EBER1* contains three Rpl22 binding sites and is thought to compete with the 28S rRNA for Rpl22 binding in Epstein-Barr virus-infected cells [42], [43], [53], [56], [57]. In yeast, recent studies have revealed regulation of the expression of one paralog in a duplicated *RP* gene pair by the other. Ribosomal protein S29A (Rps29a), for example, regulates its own expression along with expression of its paralog, *RPS29B* [58].

Interestingly, Rpl22 has also been ascribed functions independent of its role as a component of the ribosome [17], [18]; these extra-ribosomal functions include regulation of telomerase activity [44] and association with histone H1 [59]. In fact, many ribosomal proteins, including Rpl7, Rpl13a, and Rps3 [60]–[62] have extra-ribosomal functions, which will have to be considered as mechanistic links are sought to explain diseases associated with RP mutations and possibly age-related phenotypes. Given their ancient nature, it is not surprising that evolution has settled on ways of exploiting these proteins for multiple uses.

Do Rpl22 and Rpl22l1 have shared or unique functions? The observation that one ribosomal protein represses expression of its own paralog is likely to be of biological significance and determining whether mice lacking *Rpl22l1* have specific phenotypes will identify tissues or conditions where Rpl22l1 is the functional RPL22 paralog that participates in translation. We propose that Rpl22 and Rpl22l1 share the ability to participate in protein translation as part of the ribosome, since both paralogs are found incorporated into ribosomes and Rpl22 has an active role in suppressing expression of *Rpl22l1*. However, unique roles are also well established, since knockout of mouse *Rpl22* leads to specific phenotypes in T and B cell development (Anderson et al. 2007 and our data), and data from Zhang et al. indicate that both Rpl22 and Rpl22l1 are essential for T cell development in zebrafish and exhibit antagonistic functions in regulating the emergence of hematopoietic stem cells [63]. Together, these observations lead us to propose that Rpl22 and Rpl22l1 may have overlapping activities, sharing a role in enhancing large subunit ribosome function but also having distinct roles in development of the immune system [63]and, in the case of Rpl22l1, perhaps other tissues.

Based on data from *S. cerevisiae*, in which genes for most ribosomal proteins are duplicated, Komili et al. proposed the existence of a ribosome code, whereby ribosome subunits composed of different ribosomal proteins would have differential specificity for mRNAs [38]. This proposal was based on an accumulation of data, including differential localization of paralogs and large-scale phenotypic screens, which indicated that specific subsets of ribosomal protein genes were often identified in phenotypic screens of gene deletion strains. This is an exciting hypothesis that, if corroborated, would identify ribosome composition as a new mechanism for regulation of gene expression. Our studies in yeast raise the possibility that ribosome specificity may occur in yeast aging [10], [37]. In a long-lived, slow-growing and translation-compromised *rpl22aΔ* background, we find that deletion of the other paralog, *RPL22B*, causes no significant further reduction in translation, but blocks lifespan extension (Steffen et al., unpublished data). One possible explanation for this result is that the portion of ribosomes containing Rpl22b is increased in the *rpl22aΔ* background, leading to specific changes in translation conducive to enhanced replicative lifespan. Other explanations are possible and further studies will be necessary to test this important hypothesis. Structural studies do not point to an obvious mechanism by which Rpl22 paralogs would influence the pool of translated RNAs and other models involving specific non-ribosomal functions of Rpl22 and Rpl22l1 have to be identified and/or tested.

One limitation to the ribosome specificity hypothesis proposed in *S. cerevisiae* is that ribosomal proteins are generally not thought to be duplicated in other organisms. Therefore, if ribosome specificity indeed exists [38], it may be more prominent in yeast than mammals. Recently, Xue and Barna have suggested that specialized ribosomes might also regulate gene expression in mammals [64]. Our findings support the hypothesis, at least in the case of murine Rpl22, that one ribosomal protein may repress expression of the other, raising the possibility that ribosome specificity may extend to organisms other than yeast. In addition, a recent study of Rpl38 mutant embryos found that although global translation was unchanged, translation of Hox genes were altered [65], providing additional support for the hypothesis that differential composition of the ribosome might contribute to transcript-specific translational regulation. Cryo-EM studies of the eukaryotic 80S ribosome have demonstrated that Rpl38 is located on the surface of the ribosome and interacts with a region of the rRNA known as expansion segment 27 (ES27), which has two distinct orientations toward the L1 stalk or toward the exit tunnel [66], [67]. The location of Rpl38 in the ribosome is consistent with its proposed role in regulating transcript-specific translation.

The tissue specific defects observed in *Rpl22^−/−^* and *Rpl29^−/−^* mice, along with mice expressing mutated Rpl38 [65], suggest that temporal and spatial expression of RPs are critical for proper development and tissue patterning. In plants and *Drosophila*, many RPs paralogs display tissue specific variations and are differentially expressed during development [68]–[71]. In mammals, recent studies found mRNA expression patterns of RPs vary in different tissues and cell types [65], [72]. Together with the findings presented here, these studies illustrate that heterogeneous expression of RPs is tightly regulated; however additional studies will be crucial in confirming the influence of these differential expression patterns on specialized ribosome activity and message specific translation.

Further studies are also required to address the questions that have arisen from this study. Are there tissues or cell types in mice where Rpl22l1 and not Rpl22 is the predominant paralog? What is the mechanism by which Rpl22 represses expression of Rpl22l1 through interaction with its mRNA? What are the non-ribosomal functions of Rpl22 paralogs? And most importantly, do ribosomes with Rpl22l1 have different specificity for mRNAs than those with Rpl22? Findings presented in this study provide interesting leads in which to test the hypothesis that specialized ribosomes exist and, furthermore, point to a new level of regulation in ribosome biogenesis, wherein ribosomal paralogs regulate each other's synthesis to optimally maintain the organism.

## Materials and Methods

### Generation of Rpl22 null mice

A detailed description of the targeting vector along with the generation and genotyping of the *Rpl22^−/−^* mice can be found in the Text S1.

### Materials

DMEM tissue culture medium was purchased from Mediatech (Manassas, VA), L-glutamine, penicillin/streptomycin, and trypsin were from GIBCO (Carlsbad, CA). Fetal calf serum was from Gemini (West Sacramento, CA). Rpl22 antibody was from BD Biosciences (Franklin Lakes, NJ), Rpl22l1 antibody was from Santa Cruz (Santa Cruz, CA) and the Rpl7 antibody was purchased from Novus Biologicals (Littleton, CO). GAPDH antibody was from Ambion (Austin, TX). HRP-conjugated donkey anti-rabbit and donkey anti-mouse were from Jackson Immunoresearch Laboratories, Inc (West Grove, PA). Rpl22, Rpl22L1, and ARBP primers were from Operon (Huntsville, AL). Lentiviral shRNA constructs V2MM_120192 and V2LHS_131608, directed at Rpl22 (denoted shRNA 1 and 2, respectively), along with V3LMM_473587 and V3LHS_322499, directed at Rpl22l1 (denoted shRNA 1 and 2, respectively), were purchased through Open Biosystems (Huntsville, AL) and. A non-silencing-TRIPZ lentiviral inducible shRNAmir control was also purchased through Open Biosystems.

### RNA isolation and real time PCR

RNA was isolated from mouse tissue or 3T9 cells using the RNeasy kit (Qiagen) with a DNaseI treatment following the manufacturer's protocol. cDNA synthesis was performed using oligo (dT) and reverse transcriptase Superscript Preamplification System (Invitrogen). Quantitative PCR was performed using a Biorad iCycler and measuring SYBR green incorporation for product detection. The fold-increase of Rpl22 and Rpl22l1 were normalized to the housekeeping gene ARBP. The primer sequences are as follows: RPL22 (forward): 5′-GTCGCCAACAGCAAAGAGAG-3′; RPL22 (reverse): 5′-TCCTCGTCTTCCTCCTCCTC-3′; RPL22l1 (forward): 5′-TGGAGGTTTCATTTGGACCTTAC-3′; RPL22l1 (reverse): 5′-TTTCCAGTTTTTCCATTGACTTTAAC-3′. For qRT-PCR analysis of gradient fractions, values for Rpl22 and Rpl22l1 were normalized to an artificial polyadenylated RNA that was added in equal amounts to each gradient fraction.

3T9 cells were treated with 1 µM Actinomycin D for the indicated time (0, 2, 4, 6, 8 or 24 hours). Cells were washed once with PBS then total RNA was isolated and cDNA was synthesized as described above. Relative quantitation was calculated by the 2^−ΔΔCT^ method and normalized to ARBP.

### Western blot analysis

Adult mouse tissue or cultured cells were lysed in RIPA buffer (300 mM NaCl, 1.0% NP-40, 0.5% sodium deoxycholate, 0.1% SDS, 50 mM Tris pH 8.0) and proteins separated and transferred to nitrocellulose membranes using the Invitrogen (Carlsbad, CA) Nu-Page system. Proteins in polysome fractions from sucrose density gradients (described above) were TCA precipitated and resuspended in 30 ul of Sample Buffer (1× Laemmli buffer with bromophenol blue and DTT), while for mouse liver polysome fractions, 32 ul of each 1 ml fraction was loaded and proteins were separated and transferred as described above. Blots were blocked with 5% milk in TBST for 1 h and incubated overnight at 4°C with the following dilutions of primary antibodies: Rpl22 (1∶250), Rpl22l1 (1∶250), Rpl7 (1∶1000), GAPDH (1∶50,000). Membranes were incubated for 2 h with HRP-conjugated donkey anti-rabbit or sheep anti-mouse (1∶10,000) secondary antibodies and antigen was detected using enhanced chemiluminescence (ECL Plus Western Blotting Detection System, Amersham).

### Polysome analysis

Polysome analysis of mouse liver samples was adapted from the protocol of Oliver et al. [23]. Minced liver tissue was homogenized on ice using a Dounce homogenizer in ice-cold lysis buffer containing 1.5 mM KCl, 2.5 mM MgCl_2_, 5 mM Tris-HCl, pH 7.4, 1% Na deoxycholate and 1% Triton X-100. After centrifugation at 2500 g for 15 min at 4C in a microfuge, heparin and cycloheximide were added to the clarified supernatants to final concentrations of 1 mg/ml and 100 µg/ml, respectively, and centrifuged at 16,000 g for 10 min at 4°C. Samples containing 20–25 OD260 units in 1 ml were loaded onto 11 ml 7–47% (w/v) sucrose high-salt (800 mM KCl) gradients described previously [73]. Samples were centrifuged in a Beckman SW40 Ti rotor at 39,000 rpm (270,500×g) for 2 hr at 4°C and 1-ml fractions were collected from the top of the gradients with a Brandel fractionator system. The A_254_ gradient profiles shown in Figure 2 were digitized using a DATAQ DI-148U data recording module that converts and exports analog absorbance readings to analysis software.

### Mass spectrometry

A detailed description of the sample preparation and the experimental procedure can be found in the Text S1.

### MEF isolation and 3T9 cell lines

Embryos were harvested at day 13.5dpc and mouse embryonic fibroblasts (MEFs) were harvested as previously described [74]. Following isolation, MEFs were cultured in DMEM (Dulbecco's modified Eagle medium) containing 10% FBS, 1% Penicillin/Streptomycin and 1% L-Glutamine and subjected to 3T9 (Figure 6) protocol [74], [75]. Cells were removed from tissue culture plates with 0.05% trypsin and 0.02% EDTA for 3 min at 37°C, washed with DMEM, and plated overnight prior to experimentation.

### Lentiviral transduction

For Rpl22 and Rpl22l1 knockdown, lentiviral pGIPZ shRNA constructs were purchased from Open Biosystems and cloned into inducible pTRIPZ constructs. Briefly, 293T cells were transfected with pTRIPZ constructs bearing shRNA against Rpl22 (shRNA 1 and 2) or a non-silencing control using calcium phosphate transfection. After 48 h, viral supernatant was harvested and used to transduce 3T9 cells. Cells were selected for 5 days in puromycin (5 µg/ml) followed by 3 days of doxycycline (1 µg/ml) treatment to induce shRNA expression before experimentation. For cell proliferation assays, *Rpl22^+/+^* or *Rpl22^−/−^* 3T9 cells were infected with control shRNA or 1 of 2 shRNA constructs targeting Rpl22 or Rpl22l1. After 5 days of selection in puromycin, growth rates of cells transduced with each shRNA construct were determined by plating cells in triplicate at a density of 30,000 cells/well in 6-well plate in media with or without doxycycline (1 µg/ml). Every 3 days for the cells were counted and replated at 30,000 cells/well for 15 days. Protein was isolated from 10 cm plates treated in parallel to the growth assays.

### Bioinformatics

Protein sequences for murine Rpl22 (NP_033105) and Rpl22l1 (NP_080793) were obtained from Genbank (http://www.ncbi.nlm.nih.gov/). Sequence alignments were performed using ClustalW2 Algorithm (http://www.ebi.ac.uk/Tools/msa/clustalw2/). mRNA sequences for mRpl22l1 (NM_026517) and zRpl22l1 (NM_001045335) were entered into mFold (http://mfold.rna.albany.edu/?q=mfold/RNA-Folding-Form) for prediction of RNA secondary structure.

### Statistical analysis

Statistical comparisons were made between groups by a 2-tail Mann-Whitney U test (Figure 6, S5, S6), by Newman-Keuls post hoc analysis, a two-tailed Student's *t* test (Figures 4, S2, S3, S4), or a 1-way ANOVA (Figure 3). Significant differences between groups are indicated in figure legends.

### Zebrafish embryos

AB wild-type zebrafish strain was bred and maintained at 28.5°C under standard aquaculture conditions. Embryos were staged as described previously [76].

### mRNAs, morpholino microinjection and biosensor quantification assay in zebrafish embryos

Full-length coding sequence for EGFP, mCherry, zebrafish Rpl22 (zRpl22) and Rpl22l1 (zRpl22l1) were cloned into pCS2+. Full- length cDNA sequences encoding EGFP-zRpl22 and EGFP-zRpl22l1 were subcloned in the pCS2+. The zRpl22l1mut, mutant form of zRpl22l1 in which hairpin GATGGGATTCTCGATT was mutated to GACGGTATCTTAGACT, was generated using GeneTailor kit (Invitrogen, Carlsbad, CA) with the modified forward primer 5′-GACTGCACTCACCCTGTGGAGGACGGTA TCTTAGACTCT GCAAACTTTG-3′. The 150 bp fragment in the zebrafish Rpl22l1 CDS containing the Rpl22-binding hairpin was amplified by PCR using the following primers: Forward: cagggatccatgcagactgttgtgagaaagaat, Reverse : tcagaattccaggttgcctgttttgccattaac. Then the PCR product was digested (BamH1+EcoR1) and ligated into pCS2+EGFP to get the in-frame chimera sequence, which was referred to as zRpl22l1-150h-EGFP. All mRNAs for microinjection were synthesized using the mMessage mMachine kit (Ambion, Austin, TX). Then embryos were injected at one-cell stage with indicated synthetic mRNAs and observed at 10 hpf stages. Injection doses: 100 pg for each mCherry, GFP-Sensor and Inhibitor mRNAs. Morpholinos were ordered from Gene Tools (Gene Tools, LLC, Philomath, OR) and dissolved in nuclease-free water. Morpholino to bind the translation start site (AUG MOs) of zebrafish Rpl22 [63] (Sequence: 5′-CCGACAGTTTTGGCAGAAAGCCAGT-3′) was injected at 6 ng in the 1-cell stage embryos. The standard control MO from Gene Tools was used as a control (Control MO, sequence: 5′-CCTCTTACCTCAGTTACAATTTATA-3′). Images were taken from the Nikon SMZ1500 stereomicroscope equipped with DS-Fi1 digital camera and Nikon Ar imaging software.

Fluorescence sensor assays was designed and performed according to the well-established method in zebrafish [77], [78]. To calculate relative fluorescence for each embryo, green or red pixel intensity was quantified using ImageJ software (NIH). Relative fluorescence was determined for each embryo (GFP/mCherry). After normalization to the average GFP/mCherry ratio in the control embryos, the sensor repression or enhancement fold was obtained in the inhibitor mRNA or Rpl22-MO injected groups.

### Plasmids

The coding regions of EBER1 and EBER 2 were amplified by PCR from the cDNA of EBV-infected African Burkitt lymphoma KemIII cells (kindly provided by Jeffrey Sample, Penn State Hershey College of Medicine, Hershey, PA), adding a T7 promoter, and cloned into pCR2.1 plasmid (Invitrogen, Carlsbad, CA). The primers used for PCR were: forward 5′-TAATA CGACTCACTATAGGCAAAACCTCAGGACCTACGCTG, TAATACGACTCACTATAGGTCAAA CAGGACAGCCGTTGC and reverse 5′-GAACTGCGGGATAATGGATGC, AAGCCGAATACC CTTCTCCCAG for EBER 1 and EBER2 respectively. The zRpl22l1Δhp and EGFP-zRpl22l1mut were generated from plasmid zRpl22l1-pCS2+ and EGFP-zRpl22l1-pCS2+, respectively, with deletion of the hairpin TGGGATTCTCGA using GeneTailor mutagenesis kit according to manufacturer's instruction (Invitrogen, Carlsbad, CA). pGEX-m88 plasmid was generated from pGEX-hRpl22 (kindly provided by Joan Steitz, Yale University, New Haven, CT) using GeneTailor mutagenesis kit according to manufacturer's instruction (Invitrogen, Carlsbad, CA) creating a EEYLKE motif instead of KKYLKK.

### RNAse protection assay

The plasmids used for RPA assay were EBER 1, EBER 2, zRpl22l1 and zRpl22l1Δhp. The plasmids were linearized using EcoRI for EBERs and NotI for zRpl22l1s (enzymes from New England Biolab, Ipswich, MA), and radioactive probes were prepared by in vitro transcription with T7 (EBERs) or SP6 (zRpl22like1s) RNA polymerases at 37°C (Maxiscript, Ambion, TX), in presence of 10 µCi of 3ZP-UTP (Perkin Elmer, Boston, MA), and purified by G-50 columns (Illustra Probe-Quant, GE Healthcare, Buckinghamshire, UK). The radiolabeled RNAs were renatured for 2 min at 95°C and kept on ice for 5 min. Radiolabeled RNAs and protein were incubated on ice for 12 min, in presence of 5 µg of tRNA (Roche) in RPA buffer (10 mM Tris pH 8.0, 50 mM NaCl, 0.75 mM MgCl2, 0.8 mM DTT and 2.5% glycerol), and the mixtures were cross-linked on ice pack for 30 min (using a UV Stratalinker 1800). After cross-linking, the mixture were digested by RNase A (Qiagen, Valencia, CA, 7,500 U/µl) diluted to 3,750 U/µl in RNase A buffer (20 mM Tris pH 7.0, 2 mM MgCl2, and 0.2 mM KCl) for 20 min at 37°C, then 2 µl of denaturing dye were added before denaturation for 2 min at 90°C. Nine µl of the reaction mixtures were loaded on 4–12% Bis-Tris gel, 1.5 mm (Invitrogen, Carlsbad, CA). The gels were dried and expose to BioMax MR films (Kodak, Rochester, NY) or to a phosphoimager plate read using a Fuji BAS-2500 reader.

### Protein expression and purification

E. coli strain BL21DE(3)pLys (Promega, Madison, WI) was used to produce recombinant GST, GST-hRpl22 and GST-hRpl22m88 from plasmids pGEX-3X. pGEX-hRpl22 and pGEX-hRplm88. Isopropyl-β-D-thiogalactopyranoside (IPTG) at a final concentration of 100 µM was added to 2× YT media at optical density A600 = 0.4, and incubation was maintained for 4 hours at 37°C. The cells were harvested and resuspended at 0.25 mg/ml in 1× PBS (2.6 mM KCl, 1.7 mM KH2PO4, 137 mM NaCl, 11 mM Na2HPO4, pH 7.4) and lysed by 3 passages in a M-110L Pneumatic (Microfluidics, Newton, MA), in presence of Complete mini without EDTA tablets (Roche, Mannheim, Germany). The cell extracts were loaded onto glutathione sepharose beads for batch purification (GE Healthcare, Upsala, Sweden) at 2 ml/liter of culture. The beads, 0.5 ml, were washed with 1 ml of wash buffer (50 mM Tris, pH 9.5, 150 mM NaCl), then washed with 1 ml of bump buffer (50 mM Tris pH 9.5, 150 mM NaCl, 1 mM reduced GSH). The proteins were eluted with 3 fractions of 1 ml elution buffer (50 mM Tris pH 9.5, 150 mM NaCl, 10 mM reduced GSH). The protein purity was assessed by SDS gel electrophoresis, and the concentration measured using Coomassie Plus (Bradford) Protein Assay (Pierce, Rockford, IL). The proteins were stored in 10% glycerol in 20 µl aliquots at −80°C.

### Ethics statement

Appropriate protocols of all work on mice have been approved by the IACUC committees at the Institution where the work was performed and is in accord with accepted national guidelines.

## Supporting Information

Figure S1 Generation of *Rpl22^−/−^* mice. (**A**) Schematic of *Rpl22* targeting strategy; (B) Gel shows PCR of mouse tail DNA from *Rpl22^+/+^*, *Rpl22^+/−^ and Rpl22^−/−^* mice. (**C**) Western blot with α-RPL22 antibody using fibroblasts isolated from *Rpl22^+/+^* and *Rpl22* ^−/−^ mice.
(TIF)

Figure S2 *Rpl22^−/−^* mice have impaired thymocyte development. Thymocytes from *Rpl22^−/−^* mice were analyzed to confirm that *Rpl22* deficiency resulted in altered T cell development. Thymocytes were stained for CD4, CD8, CD44, CD25, panTCRβ, and TCRγδ surface expression. (**A**) Representative flow cytometry plots show CD4/CD8 analysis of live-gated thymocytes. Charts show the absolute number of each indicated population. The thymic compartment in *Rpl22^−/−^* mice was comprised mostly of CD4−CD8− double negative (DN) thymocytes, while CD4+CD8+ double-positive (DP) and CD4 and CD8 single-positive (SP) populations were clearly diminished. Thymic cellularity was significantly reduced at the DN, DP, and SP stages. (**B**) CD44/CD25 plots of CD4−CD8− DN thymocytes and chart showing the absolute number of each DN population (DN1: CD44+CD25−, DN2: CD44+CD25+, DN3: CD44−CD25+, DN4: CD44−CD25−). DN thymocytes had an apparent blockade of development between the DN3 (CD44−CD25+) to DN4 (CD44−CD25−) stages, as evidenced by an increased frequency of DN3 cells, and significantly reduced frequency and number of DN4 cells. (**C**) Representative flow cytometry plots of total thymocytes analyzed for TCRγδ and panTCRβ surface expression. Chart shows the absolute number of TCRγδ+ thymocytes. γδ T cell development was unimpaired, as evidenced by the similar number of TCR γδ + cells in the thymus of *Rpl22^−/−^* mice. Data are compiled from 3 independent experiments (*Rpl22^+/+^* N = 4, *Rpl22* ^−/−^ N = 4). Numbers in plots represent the percentage of each gated population. Charts show the mean percent or number with error bars indicating the standard deviation. P values were calculated using a two-tailed Student's *t* test (* p<0.05, **p<0.001, ***p<0.0001).
(TIF)

Figure S3 αβ T cell and B cell numbers are reduced in the lymphoid periphery of *Rpl22^−/−^* mice. Splenocytes were surface stained for CD4, CD8, CD19, panTCRβ, and TCRγδ. (**A**) Representative flow cytometry plots and chart show the percent of CD19+ B cells and CD4+ and CD8+ T cells. (**B**) Absolute number of total splenocytes and CD19+, CD4+ and CD8+ cells per spleen. (**A,B**) *Rpl22^−/−^* mice also had reduced frequencies and numbers of splenic CD4+ and CD8+ T cells and reduced B cell numbers. (**C**) Representative plots and chart showing percent of TCRβ+ and TCRγδ+ cells per spleen. (**D**) Absolute number of TCRβ+ and TCRγδ+ cells per spleen. The frequency of splenic γδ T cells was significantly increased (**C**), while the γδ T cell number remained similar to that of wild-type mice (**D**). Data are compiled from 2 independent experiments (*Rpl22^+/+^* N = 3, *Rpl22^−/−^* N = 3). Numbers in plots represent the percentage of each gated population. Charts show the mean percent or number with error bars indicating the standard deviation. P values were calculated using a two-tailed Student's *t* test (* p<0.05, ** p<0.001, *** p<0.0001).
(TIF)

Figure S4 B cell development in *Rpl22^−/−^* mice is impaired. Bone marrow single-cell suspensions were stained for surface B220, IgM, and IgD. (**A**) Representative histograms show the percent of live-gated cells that are B220+. (**B**) Absolute number of total bone marrow cells and B220− and B220+ populations. (**C**) Representative IgM/IgD plots of B220+ gated bone marrow cells. (**D**) Absolute number of B220+ bone marrow cells that are IgM−IgD−, IgM+IgD− (immature B cells), and IgM+IgD+ (mature B cells). Data are compiled from 2 independent experiments (*Rpl22^+/+^* N = 3, *Rpl22^−/−^* N = 3). Numbers in plots represent the percentage of each gated population. Charts show the mean percent or number with error bars indicating the standard deviation. P values were calculated using a two-tailed Student's *t* test (** p<0.001).
(TIF)

Figure S5 Alignment of mouse, human and zebrafish *Rpl22l1* protein sequences. Identical amino acids are indicated by an * beneath the alignment.
(TIF)

Figure S6 Knockdown of Rpl22l1 expression with a second shRNA impairs cellular growth. *Rpl22^−/−^* 3T9 cells were transduced with a second shRNA construct directed at Rpl22l1 (shRNA2-Rpl22l1) and selected with puromycin for at least 5 days. Growth rates of cells transduced with the shRNA construct were determined by plating cells in triplicate at a density of 30,000 cells/well in 6-well plate in media with or without doxycycline (1 µg/ml). Every 3 days for the cells were counted and replated at 30,000 cells/well for 15 days. (**A**) Growth of *Rpl22^−/−^* 3T9 cells transduced with the shRNA#2-Rpl22l1 construct was determined. Knockdown of Rpl22l1 by shRNA#2-Rpl22l1 represses growth in *Rpl22^−/−^* cells. (**B**) Levels of Rpl22l1 were analyzed by Western blot analysis to confirm that the Rpl22l1-shRNA knocked down levels of Rpl22l1 in doxycycline-treated *Rpl22^−/−^* 3T9 cells transduced with the shRNA#2-Rpl22l1 construct. Results are representative of 2 independent experiments with error bars representative of ±SD. Statistical significance is indicated (*, p<0.05 compared to untreated control).
(TIF)

Figure S7 Acute knockdown of Rpl22 expression has no significant effect on cellular growth. *Rpl22^+/+^* 3T9 cells were transduced with doxycycline-inducible shRNA lentiviral constructs directed at Rpl22 (shRNA1-Rpl22 or shRNA2-Rpl22) or a non-specific shRNA construct (shRNA-NS) and selected with puromycin for at least 5 days. Growth rates of cells transduced with the shRNA construct were determined by plating cells in triplicate at a density of 30,000 cells/well in 6-well plate in media with or without doxycycline (1 µg/ml). Every 3 days for the cells were counted and replated at 30,000 cells/well for 15 days. Growth of *Rpl22^+/+^* 3T9 cells transduced with each shRNA construct was determined. Knockdown of Rpl22 by (**A**) shRNA1-Rpl22 or (**B**) shRNA2-Rpl22 does not repress growth in *Rpl22^+/+^* cells. Results are representative of 2 independent experiments with error bars representative of ±SD.
(TIF)

Text S1 Supplementary text extending Materials and Methods.
(DOCX)

## References

[1] Planta RJ (1997) Regulation of ribosome synthesis in yeast. Yeast 13: 1505–1518. 9509571 10.1002/(SICI)1097-0061(199712)13:16<1505::AID-YEA229>3.0.CO;2-I

[2] Rudra D, Warner JR (2004) What better measure than ribosome synthesis? Genes Dev 18: 2431–2436. 15489289 10.1101/gad.1256704

[3] Laferte A, Favry E, Sentenac A, Riva M, Carles C, et al (2006) The transcriptional activity of RNA polymerase I is a key determinant for the level of all ribosome components. Genes Dev 20: 2030–2040. 16882981 10.1101/gad.386106 PMC1536055

[4] Fromont-Racine M, Senger B, Saveanu C, Fasiolo F (2003) Ribosome assembly in eukaryotes. Gene 313: 17–42. 12957375 10.1016/s0378-1119(03)00629-2

[5] Liu JM, Ellis SR (2006) Ribosomes and marrow failure: coincidental association or molecular paradigm? Blood 107: 4583–4588. 16507776 10.1182/blood-2005-12-4831

[6] Narla A, Ebert BL (2010) Ribosomopathies: human disorders of ribosome dysfunction. Blood 115: 3196–3205. 20194897 10.1182/blood-2009-10-178129 PMC2858486

[7] Scheper GC, van der Knaap MS, Proud CG (2007) Translation matters: protein synthesis defects in inherited disease. Nat Rev Genet 8: 711–723. 17680008 10.1038/nrg2142

[8] Ellis SR, Lipton JM (2008) Diamond Blackfan anemia: a disorder of red blood cell development. Curr Top Dev Biol 82: 217–241. 18282522 10.1016/S0070-2153(07)00008-7

[9] Cmejla R, Cmejlova J, Handrkova H, Petrak J, Pospisilova D (2007) Ribosomal protein S17 gene (RPS17) is mutated in Diamond-Blackfan anemia. Hum Mutat 28: 1178–1182. 17647292 10.1002/humu.20608

[10] Steffen KK, MacKay VL, Kerr EO, Tsuchiya M, Hu D, et al (2008) Yeast life span extension by depletion of 60s ribosomal subunits is mediated by Gcn4. Cell 133: 292–302. 18423200 10.1016/j.cell.2008.02.037 PMC2749658

[11] Chiocchetti A, Zhou J, Zhu H, Karl T, Haubenreisser O, et al (2007) Ribosomal proteins Rpl10 and Rps6 are potent regulators of yeast replicative life span. Exp Gerontol 42: 275–286. 17174052 10.1016/j.exger.2006.11.002

[12] Hansen M, Taubert S, Crawford D, Libina N, Lee SJ, et al (2007) Lifespan extension by conditions that inhibit translation in Caenorhabditis elegans. Aging Cell 6: 95–110. 17266679 10.1111/j.1474-9726.2006.00267.x

[13] Kaeberlein M, Powers RW (2005) Regulation of yeast replicative life span by TOR and Sch9 in response to nutrients. Science 310: 1193–1196. 16293764 10.1126/science.1115535

[14] Brodersen DE, Nissen P (2005) The social life of ribosomal proteins. Febs J 272: 2098–2108. 15853795 10.1111/j.1742-4658.2005.04651.x

[15] Noller HF, Hoffarth V, Zimniak L (1992) Unusual resistance of peptidyl transferase to protein extraction procedures. Science 256: 1416–1419. 1604315 10.1126/science.1604315

[16] Wool IG (1996) Extraribosomal functions of ribosomal proteins. Trends Biochem Sci 21: 164–165. 8871397

[17] Warner JR, McIntosh KB (2009) How common are extraribosomal functions of ribosomal proteins? Molecular cell 34: 3–11. 19362532 10.1016/j.molcel.2009.03.006 PMC2679180

[18] Bhavsar RB, Makley LN, Tsonis PA (2010) The other lives of ribosomal proteins. Human genomics 4: 327–344. 20650820 10.1186/1479-7364-4-5-327 PMC3500163

[19] Vilardell J, Warner JR (1994) Regulation of splicing at an intermediate step in the formation of the spliceosome. Genes & development 8: 211–220. 8299940 10.1101/gad.8.2.211

[20] Macias S, Bragulat M, Tardiff DF, Vilardell J (2008) L30 binds the nascent RPL30 transcript to repress U2 snRNP recruitment. Molecular cell 30: 732–742. 18570876 10.1016/j.molcel.2008.05.002

[21] Malygin AA, Parakhnevitch NM, Ivanov AV, Eperon IC, Karpova GG (2007) Human ribosomal protein S13 regulates expression of its own gene at the splicing step by a feedback mechanism. Nucleic acids research 35: 6414–6423. 17881366 10.1093/nar/gkm701 PMC2095825

[22] Matsson H, Davey EJ, Draptchinskaia N, Hamaguchi I, Ooka A, et al (2004) Targeted disruption of the ribosomal protein S19 gene is lethal prior to implantation. Mol Cell Biol 24: 4032–4037. 15082795 10.1128/MCB.24.9.4032-4037.2004 PMC387766

[23] Oliver ER, Saunders TL, Tarle SA, Glaser T (2004) Ribosomal protein L24 defect in belly spot and tail (Bst), a mouse Minute. Development 131: 3907–3920. 15289434 10.1242/dev.01268 PMC2262800

[24] Tang Q, Rice DS, Goldowitz D (1999) Disrupted retinal development in the embryonic belly spot and tail mutant mouse. Developmental biology 207: 239–255. 10049578 10.1006/dbio.1998.9142

[25] Ruvinsky I, Sharon N, Lerer T, Cohen H, Stolovich-Rain M, et al (2005) Ribosomal protein S6 phosphorylation is a determinant of cell size and glucose homeostasis. Genes & development 19: 2199–2211. 16166381 10.1101/gad.351605 PMC1221890

[26] Volarevic S, Stewart MJ, Ledermann B, Zilberman F, Terracciano L, et al (2000) Proliferation, but not growth, blocked by conditional deletion of 40S ribosomal protein S6. Science 288: 2045–2047. 10856218 10.1126/science.288.5473.2045

[27] Panic L, Tamarut S, Sticker-Jantscheff M, Barkic M, Solter D, et al (2006) Ribosomal protein S6 gene haploinsufficiency is associated with activation of a p53-dependent checkpoint during gastrulation. Molecular and cellular biology 26: 8880–8891. 17000767 10.1128/MCB.00751-06 PMC1636830

[28] Anderson SJ, Lauritsen JP, Hartman MG, Foushee AM, Lefebvre JM, et al (2007) Ablation of ribosomal protein L22 selectively impairs alphabeta T cell development by activation of a p53-dependent checkpoint. Immunity 26: 759–772. 17555992 10.1016/j.immuni.2007.04.012

[29] Kirn-Safran CB, Oristian DS, Focht RJ, Parker SG, Vivian JL, et al (2007) Global growth deficiencies in mice lacking the ribosomal protein HIP/RPL29. Dev Dyn 236: 447–460. 17195189 10.1002/dvdy.21046

[30] Wolfe KH, Shields DC (1997) Molecular evidence for an ancient duplication of the entire yeast genome. Nature 387: 708–713. 9192896 10.1038/42711

[31] Rotenberg MO, Moritz M, Woolford JL Jr (1988) Depletion of Saccharomyces cerevisiae ribosomal protein L16 causes a decrease in 60S ribosomal subunits and formation of half-mer polyribosomes. Genes Dev 2: 160–172. 3282992 10.1101/gad.2.2.160

[32] Dean EJ, Davis JC, Davis RW, Petrov DA (2008) Pervasive and persistent redundancy among duplicated genes in yeast. PLoS genetics 4: e1000113. 18604285 10.1371/journal.pgen.1000113 PMC2440806

[33] Baudin-Baillieu A, Tollervey D, Cullin C, Lacroute F (1997) Functional analysis of Rrp7p, an essential yeast protein involved in pre-rRNA processing and ribosome assembly. Mol Cell Biol 17: 5023–5032. 9271380 10.1128/mcb.17.9.5023 PMC232353

[34] Haarer B, Viggiano S, Hibbs MA, Troyanskaya OG, Amberg DC (2007) Modeling complex genetic interactions in a simple eukaryotic genome: actin displays a rich spectrum of complex haploinsufficiencies. Genes Dev 21: 148–159. 17167106 10.1101/gad.1477507 PMC1770898

[35] Ni L, Snyder M (2001) A genomic study of the bipolar bud site selection pattern in Saccharomyces cerevisiae. Mol Biol Cell 12: 2147–2170. 11452010 10.1091/mbc.12.7.2147 PMC55669

[36] Enyenihi AH, Saunders WS (2003) Large-scale functional genomic analysis of sporulation and meiosis in Saccharomyces cerevisiae. Genetics 163: 47–54. 12586695 10.1093/genetics/163.1.47 PMC1462418

[37] Steffen KK, McCormick MA, Pham KM, MacKay VL, Delaney JR, et al (2012) Ribosome deficiency protects against ER stress in Saccharomyces cerevisiae. Genetics 191: 107–118. 22377630 10.1534/genetics.111.136549 PMC3338253

[38] Komili S, Farny NG, Roth FP, Silver PA (2007) Functional specificity among ribosomal proteins regulates gene expression. Cell 131: 557–571. 17981122 10.1016/j.cell.2007.08.037 PMC2443060

[39] Costanzo M, Baryshnikova A, Bellay J, Kim Y, Spear ED, et al (2010) The genetic landscape of a cell. Science 327: 425–431. 20093466 10.1126/science.1180823 PMC5600254

[40] Auger-Buendia MA, Longuet M, Tavitian A (1979) Kinetic studies on ribosomal proteins assembly in preribosomal particles and ribosomal subunits of mammalian cells. Biochim Biophys Acta 563: 113–128. 497202 10.1016/0005-2787(79)90012-1

[41] Lavergne JP, Conquet F, Reboud JP, Reboud AM (1987) Role of acidic phosphoproteins in the partial reconstitution of the active 60 S ribosomal subunit. FEBS Lett 216: 83–88. 3582668 10.1016/0014-5793(87)80761-5

[42] Dobbelstein M, Shenk T (1995) In vitro selection of RNA ligands for the ribosomal L22 protein associated with Epstein-Barr virus-expressed RNA by using randomized and cDNA-derived RNA libraries. J Virol 69: 8027–8034. 7494316 10.1128/jvi.69.12.8027-8034.1995 PMC189748

[43] Toczyski DP, Matera AG, Ward DC, Steitz JA (1994) The Epstein-Barr virus (EBV) small RNA EBER1 binds and relocalizes ribosomal protein L22 in EBV-infected human B lymphocytes. Proc Natl Acad Sci U S A 91: 3463–3467. 8159770 10.1073/pnas.91.8.3463 PMC43597

[44] Le S, Sternglanz R, Greider CW (2000) Identification of two RNA-binding proteins associated with human telomerase RNA. Mol Biol Cell 11: 999–1010. 10712515 10.1091/mbc.11.3.999 PMC14826

[45] Rao S, Lee SY, Gutierrez A, Perrigoue J, Thapa RJ, et al (2012) Inactivation of ribosomal protein L22 promotes transformation by induction of the stemness factor, Lin28B. Blood 120: 3764–3773. 22976955 10.1182/blood-2012-03-415349 PMC3488889

[46] Finak G, Bertos N, Pepin F, Sadekova S, Souleimanova M, et al (2008) Stromal gene expression predicts clinical outcome in breast cancer. Nature medicine 14: 518–527. 10.1038/nm1764 18438415

[47] Bhattacharjee A, Richards WG, Staunton J, Li C, Monti S, et al (2001) Classification of human lung carcinomas by mRNA expression profiling reveals distinct adenocarcinoma subclasses. Proceedings of the National Academy of Sciences of the United States of America 98: 13790–13795. 11707567 10.1073/pnas.191502998 PMC61120

[48] Ballif BA, Cao Z, Schwartz D, Carraway KL (2006) Identification of 14-3-3epsilon substrates from embryonic murine brain. J Proteome Res 5: 2372–2379. 16944949 10.1021/pr060206k

[49] Sugihara Y, Honda H, Iida T, Morinaga T, Hino S, et al (2010) Proteomic analysis of rodent ribosomes revealed heterogeneity including ribosomal proteins L10-like, L22-like 1, and L39-like. Journal of proteome research 9: 1351–1366. 20063902 10.1021/pr9008964

[50] Keel SB, Phelps S, Sabo KM, O'Leary MN, Kirn-Safran CB, et al (2012) Establishing Rps6 hemizygous mice as a model for studying how ribosomal protein haploinsufficiency impairs erythropoiesis. Experimental hematology 40: 290–294. 22198155 10.1016/j.exphem.2011.12.003 PMC3319152

[51] Giaever G, Chu AM, Ni L, Connelly C, Riles L, et al (2002) Functional profiling of the *Saccharomyces cerevisiae* genome. Nature 418: 387–391. 12140549 10.1038/nature00935

[52] Bensaude O (2011) Inhibiting eukaryotic transcription: Which compound to choose? How to evaluate its activity? Transcription 2: 103–108. 21922053 10.4161/trns.2.3.16172 PMC3173647

[53] Toczyski DP, Steitz JA (1993) The cellular RNA-binding protein EAP recognizes a conserved stem-loop in the Epstein-Barr virus small RNA EBER 1. Molecular and cellular biology 13: 703–710. 8380232 10.1128/mcb.13.1.703-710.1993 PMC358948

[54] Zuker M (2003) Mfold web server for nucleic acid folding and hybridization prediction. Nucleic acids research 31: 3406–3415. 12824337 10.1093/nar/gkg595 PMC169194

[55] Liang H, Li WH (2009) Functional compensation by duplicated genes in mouse. Trends in genetics : TIG 25: 441–442. 19783063 10.1016/j.tig.2009.08.001 PMC2764834

[56] Fok V, Mitton-Fry RM, Grech A, Steitz JA (2006) Multiple domains of EBER 1, an Epstein-Barr virus noncoding RNA, recruit human ribosomal protein L22. RNA 12: 872–882. 16556938 10.1261/rna.2339606 PMC1440895

[57] Houmani JL, Davis CI, Ruf IK (2009) Growth-promoting properties of Epstein-Barr virus EBER-1 RNA correlate with ribosomal protein L22 binding. Journal of virology 83: 9844–9853. 19640998 10.1128/JVI.01014-09 PMC2747990

[58] Parenteau J, Durand M, Morin G, Gagnon J, Lucier JF, et al (2011) Introns within ribosomal protein genes regulate the production and function of yeast ribosomes. Cell 147: 320–331. 22000012 10.1016/j.cell.2011.08.044

[59] Ni JQ, Liu LP, Hess D, Rietdorf J, Sun FL (2006) Drosophila ribosomal proteins are associated with linker histone H1 and suppress gene transcription. Genes Dev 20: 1959–1973. 16816001 10.1101/gad.390106 PMC1522087

[60] Kim J, Chubatsu LS, Admon A, Stahl J, Fellous R, et al (1995) Implication of mammalian ribosomal protein S3 in the processing of DNA damage. The Journal of biological chemistry 270: 13620–13629. 7775413 10.1074/jbc.270.23.13620

[61] Kuhn JF, Tran EJ, Maxwell ES (2002) Archaeal ribosomal protein L7 is a functional homolog of the eukaryotic 15.5 kD/Snu13p snoRNP core protein. Nucleic acids research 30: 931–941. 11842104 10.1093/nar/30.4.931 PMC100351

[62] Mazumder B, Sampath P, Seshadri V, Maitra RK, DiCorleto PE, et al (2003) Regulated release of L13a from the 60S ribosomal subunit as a mechanism of transcript-specific translational control. Cell 115: 187–198. 14567916 10.1016/s0092-8674(03)00773-6 PMC13188775

[63] Zhang Y, Duc AC, Rao S, Sun XL, Bilbee AN, et al (2013) Control of hematopoietic stem cell emergence by antagonistic functions of ribosomal protein paralogs. Developmental cell 24: 411–425. 23449473 10.1016/j.devcel.2013.01.018 PMC3586312

[64] Xue S, Barna M (2012) Specialized ribosomes: a new frontier in gene regulation and organismal biology. Nature reviews Molecular cell biology 13: 355–369. 22617470 10.1038/nrm3359 PMC4039366

[65] Kondrashov N, Pusic A, Stumpf CR, Shimizu K, Hsieh AC, et al (2011) Ribosome-mediated specificity in Hox mRNA translation and vertebrate tissue patterning. Cell 145: 383–397. 21529712 10.1016/j.cell.2011.03.028 PMC4445650

[66] Armache JP, Jarasch A, Anger AM, Villa E, Becker T, et al (2010) Cryo-EM structure and rRNA model of a translating eukaryotic 80S ribosome at 5.5-A resolution. Proceedings of the National Academy of Sciences of the United States of America 107: 19748–19753. 20980660 10.1073/pnas.1009999107 PMC2993355

[67] Armache JP, Jarasch A, Anger AM, Villa E, Becker T, et al (2010) Localization of eukaryote-specific ribosomal proteins in a 5.5-A cryo-EM map of the 80S eukaryotic ribosome. Proceedings of the National Academy of Sciences of the United States of America 107: 19754–19759. 20974910 10.1073/pnas.1010005107 PMC2993421

[68] Marygold SJ, Roote J, Reuter G, Lambertsson A, Ashburner M, et al (2007) The ribosomal protein genes and Minute loci of Drosophila melanogaster. Genome biology 8: R216. 17927810 10.1186/gb-2007-8-10-r216 PMC2246290

[69] Weijers D, Franke-van Dijk M, Vencken RJ, Quint A, Hooykaas P, et al (2001) An Arabidopsis Minute-like phenotype caused by a semi-dominant mutation in a RIBOSOMAL PROTEIN S5 gene. Development 128: 4289–4299. 11684664 10.1242/dev.128.21.4289

[70] Degenhardt RF, Bonham-Smith PC (2008) Transcript profiling demonstrates absence of dosage compensation in Arabidopsis following loss of a single RPL23a paralog. Planta 228: 627–640. 18566829 10.1007/s00425-008-0765-6

[71] Williams ME, Sussex IM (1995) Developmental regulation of ribosomal protein L16 genes in Arabidopsis thaliana. The Plant journal : for cell and molecular biology 8: 65–76. 7655508 10.1046/j.1365-313x.1995.08010065.x

[72] Bortoluzzi S, d'Alessi F, Romualdi C, Danieli GA (2001) Differential expression of genes coding for ribosomal proteins in different human tissues. Bioinformatics 17: 1152–1157. 11751223 10.1093/bioinformatics/17.12.1152

[73] MacKay VL, Li X, Flory MR, Turcott E, Law GL, et al (2004) Gene expression analyzed by high-resolution state array analysis and quantitative proteomics: response of yeast to mating pheromone. Mol Cell Proteomics 3: 478–489. 14766929 10.1074/mcp.M300129-MCP200

[74] Kamijo T, Zindy F, Roussel MF, Quelle DE, Downing JR, et al (1997) Tumor suppression at the mouse INK4a locus mediated by the alternative reading frame product p19ARF. Cell 91: 649–659. 9393858 10.1016/s0092-8674(00)80452-3

[75] Todaro GJ, Green H (1963) Quantitative studies of the growth of mouse embryo cells in culture and their development into established lines. The Journal of cell biology 17: 299–313. 13985244 10.1083/jcb.17.2.299 PMC2106200

[76] Kimmel CB, Ballard WW, Kimmel SR, Ullmann B, Schilling TF (1995) Stages of embryonic development of the zebrafish. Developmental dynamics : an official publication of the American Association of Anatomists 203: 253–310. 8589427 10.1002/aja.1002030302

[77] Giraldez AJ, Mishima Y, Rihel J, Grocock RJ, Van Dongen S, et al (2006) Zebrafish MiR-430 promotes deadenylation and clearance of maternal mRNAs. Science 312: 75–79. 16484454 10.1126/science.1122689

[78] Staton AA, Knaut H, Giraldez AJ (2011) miRNA regulation of Sdf1 chemokine signaling provides genetic robustness to germ cell migration. Nature genetics 43: 204–211. 21258340 10.1038/ng.758 PMC3071589

[79] Martin TE (1973) A simple general method to determine the proportion of active ribosomes in eukaryotic cells. Experimental cell research 80: 496–498. 4745392 10.1016/0014-4827(73)90333-9

