# Supplementary figures and images for: The Ribosomal Protein Rpl22 Controls Ribosome Composition by Directly Repressing Expression of Its Own Paralog, Rpl22l1
Source: PLoS Genet. 2013 Aug 22;9(8):e1003708. doi: 10.1371/journal.pgen.1003708 (PMC3750023; doi:10.1371/journal.pgen.1003708)

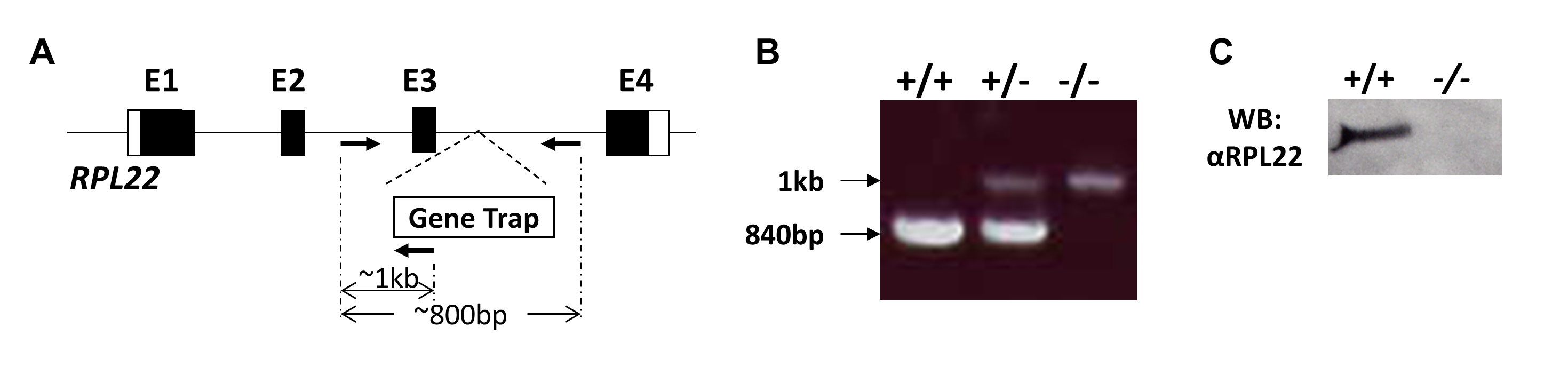

Supplement: Figure S1 — Generation of Rpl22−/− mice. (A) Schematic of Rpl22 targeting strategy; (B) Gel shows PCR of mouse tail DNA from Rpl22+/+, Rpl22+/− and Rpl22−/− mice. (C) Western blot with α-RPL22 antibody using fibroblasts isolated from Rpl22+/+ and Rpl22 −/− mice. (TIF) [file pgen.1003708.s001.tif]

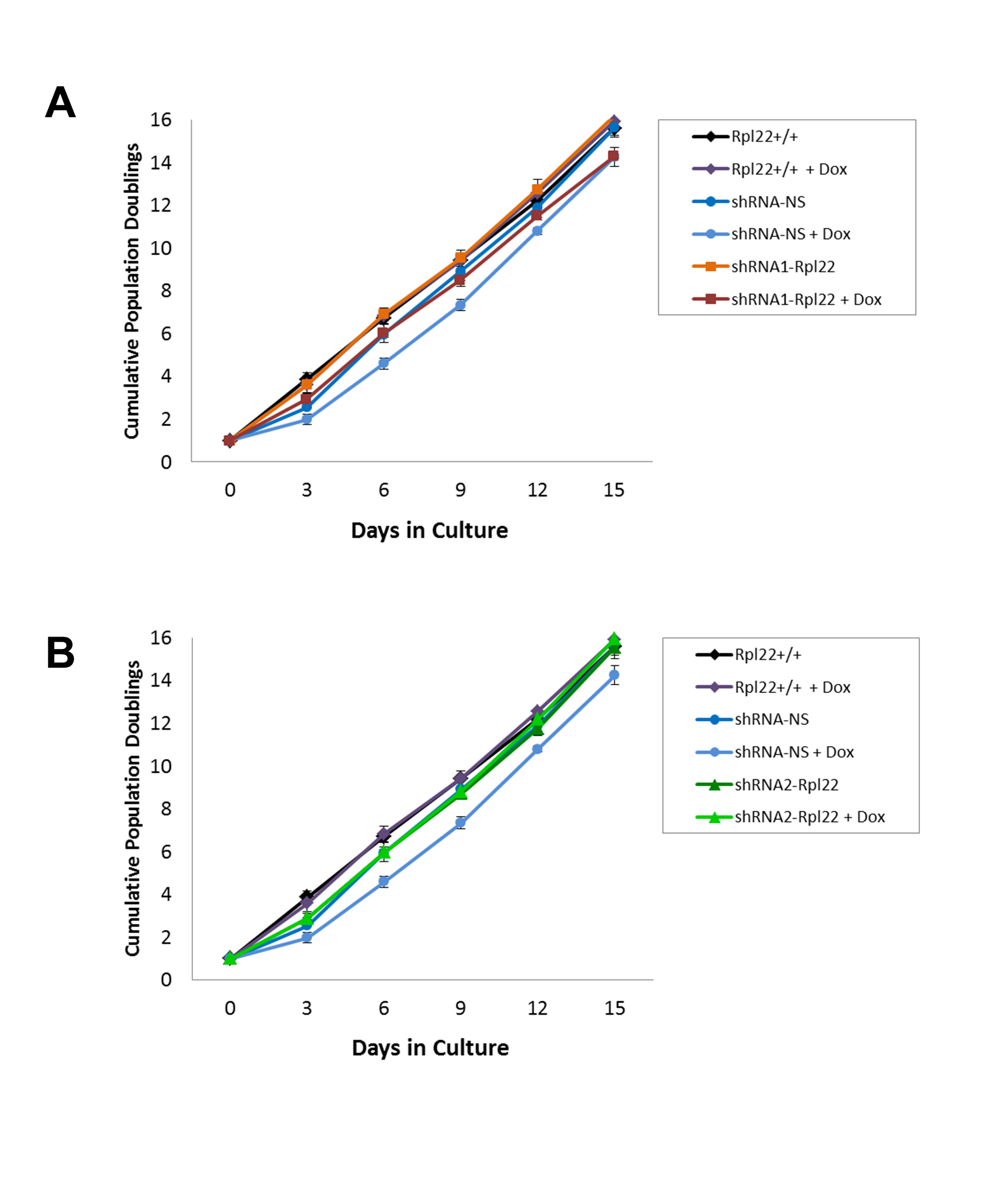

Supplement: Figure S5 — Alignment of mouse, human and zebrafish Rpl22l1 protein sequences. Identical amino acids are indicated by an * beneath the alignment. (TIF) [file pgen.1003708.s005.tif]

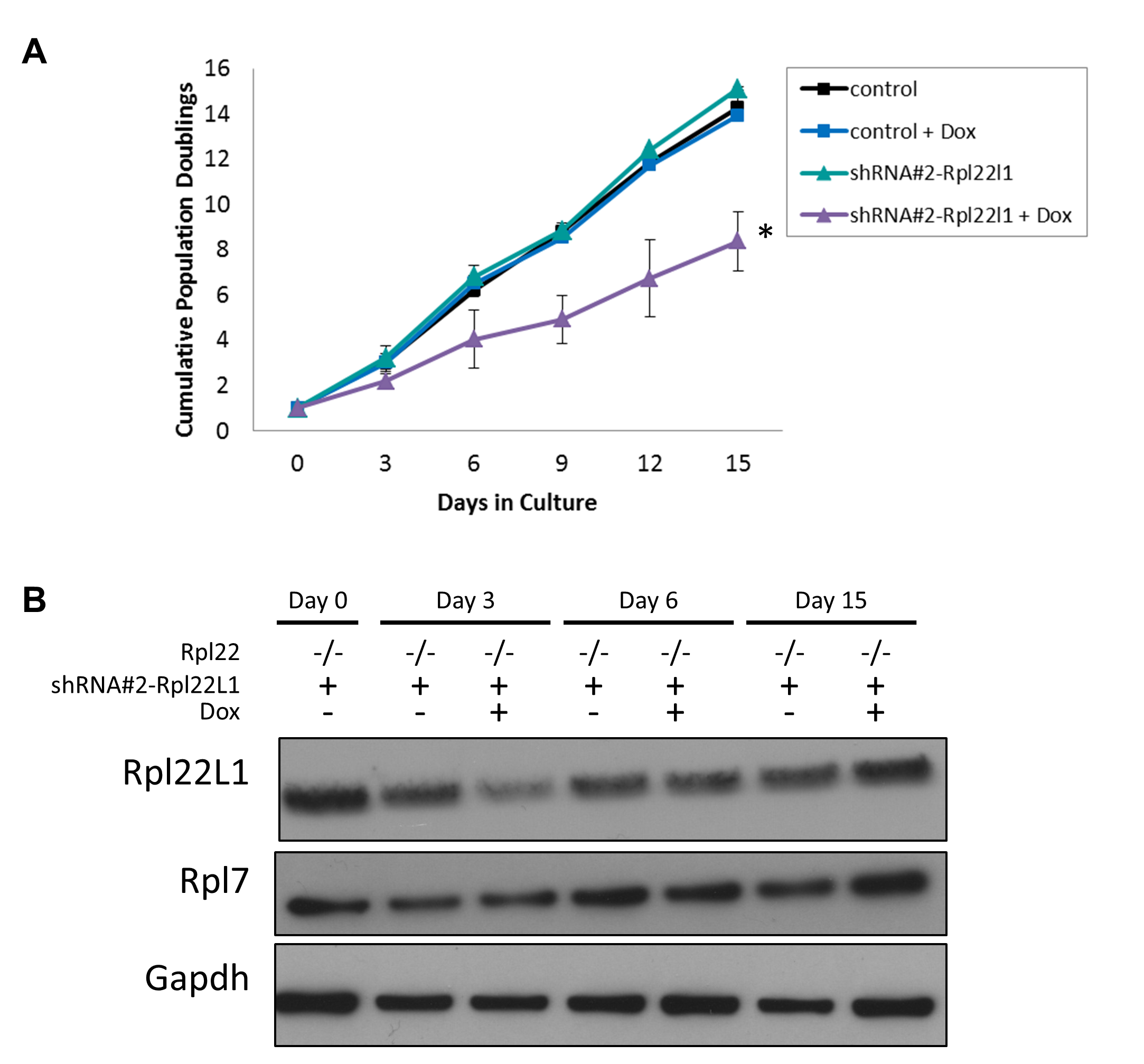

Supplement: Figure S6 — Knockdown of Rpl22l1 expression with a second shRNA impairs cellular growth. Rpl22−/− 3T9 cells were transduced with a second shRNA construct directed at Rpl22l1 (shRNA2-Rpl22l1) and selected with puromycin for at least 5 days. Growth rates of cells transduced with the shRNA construct were determined by plating cells in triplicate at a density of 30,000 cells/well in 6-well plate in media with or without doxycycline (1 µg/ml). Every 3 days for the cells were counted and replated at 30,000 cells/well for 15 days. (A) Growth of Rpl22−/− 3T9 cells transduced with the shRNA#2-Rpl22l1 construct was determined. Knockdown of Rpl22l1 by shRNA#2-Rpl22l1 represses growth in Rpl22−/− cells. (B) Levels of Rpl22l1 were analyzed by Western blot analysis to confirm that the Rpl22l1-shRNA knocked down levels of Rpl22l1 in doxycycline-treated Rpl22−/− 3T9 cells transduced with the shRNA#2-Rpl22l1 construct. Results are representative of 2 independent experiments with error bars representative of ±SD. Statistical significance is indicated (*, p<0.05 compared to untreated control). (TIF) [file pgen.1003708.s006.tif]

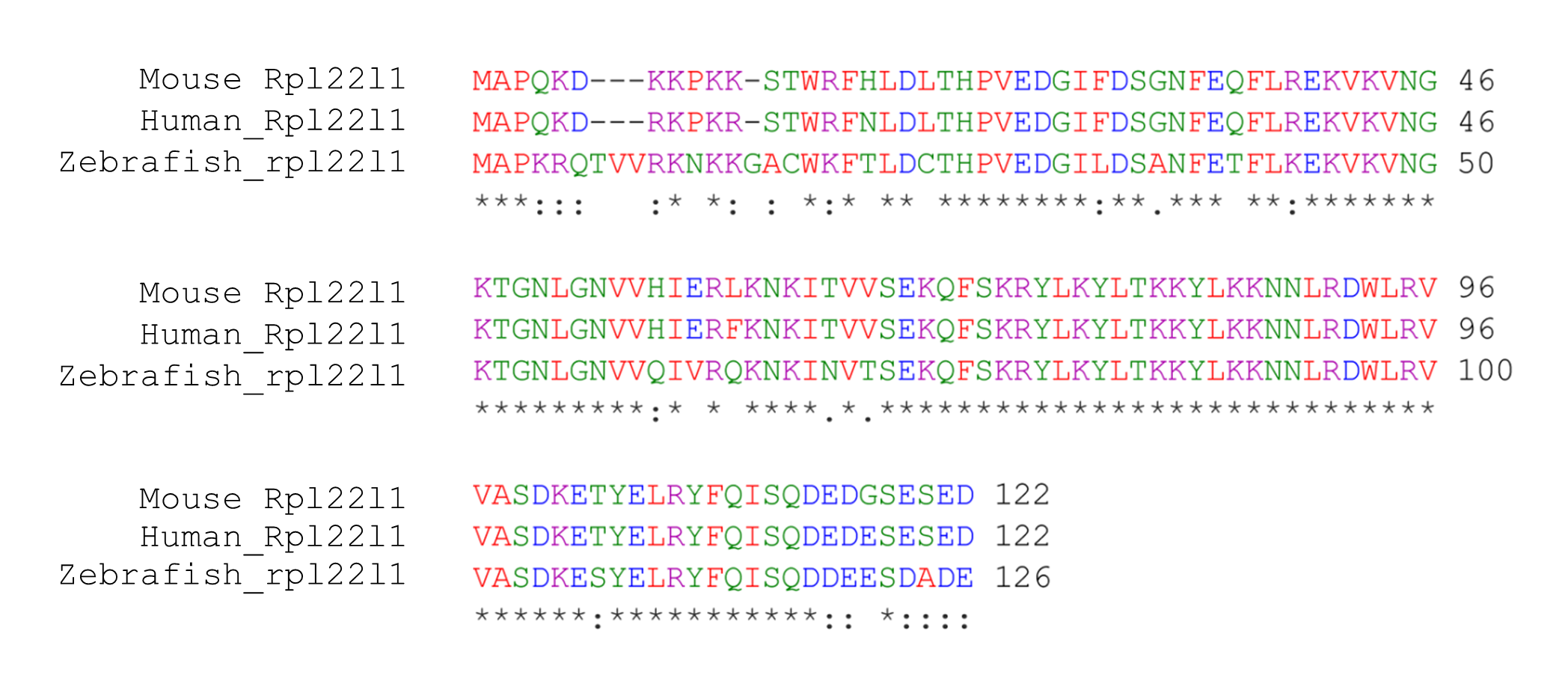

Supplement: Figure S7 — Acute knockdown of Rpl22 expression has no significant effect on cellular growth. Rpl22+/+ 3T9 cells were transduced with doxycycline-inducible shRNA lentiviral constructs directed at Rpl22 (shRNA1-Rpl22 or shRNA2-Rpl22) or a non-specific shRNA construct (shRNA-NS) and selected with puromycin for at least 5 days. Growth rates of cells transduced with the shRNA construct were determined by plating cells in triplicate at a density of 30,000 cells/well in 6-well plate in media with or without doxycycline (1 µg/ml). Every 3 days for the cells were counted and replated at 30,000 cells/well for 15 days. Growth of Rpl22+/+ 3T9 cells transduced with each shRNA construct was determined. Knockdown of Rpl22 by (A) shRNA1-Rpl22 or (B) shRNA2-Rpl22 does not repress growth in Rpl22+/+ cells. Results are representative of 2 independent experiments with error bars representative of ±SD. (TIF) [file pgen.1003708.s007.tif]
