# Supplementary material for: The Ribosomal Protein Rpl22 Controls Ribosome Composition by Directly Repressing Expression of Its Own Paralog, Rpl22l1
Source: PLoS Genet. 2013 Aug 22;9(8):e1003708. doi: 10.1371/journal.pgen.1003708 (PMC3750023; doi:10.1371/journal.pgen.1003708)
Supplement: Text S1 — Supplementary text extending Materials and Methods. (DOCX) [file pgen.1003708.s008.docx]

**Supplemental Experimental Procedures**

**Generation of Rpl22 Null Mice:** A 129 Ola embryonic stem (ES) cell line (clone ID# YHB332) that contained a mutation disrupting *Rpl22* expression was obtained from Bay Genomics (now available through the International Gene Trap Consortium. 5’ RACE and automated DNA sequencing determined that the gene-trap vector inserted between the third and fourth exons of *Rpl22* (*Rpl22*, Accession number NM_009079). This was confirmed by additional sequencing and PCR analysis. The mutant ES cell clone YHB332 was injected into mouse blastocysts to generate chimeras. A male chimera was identified by coat color to be carrying the correct ES cell contribution and bred with C57BL/6 females. Offspring were shown by PCR to carry the insertional gene-trap mutation in *Rpl22*, confirming germ line transmission of the mutation. A founder was selected to breed with C57BL/6 females to expand the line. PCR genotyping made use of the following oligonucleotide primers: (F) 5’- CCA TGG CAC ACG GAC CCA CA -3’; (WT-R) 5’-GCC TCA GCC CAC AGG CTC AC-3’; (KO-R) 5’- CGC CAG GGT TTT CCC AGT CA -3’, in a multiplex reaction on mouse tail DNA. All animal care and experimental procedures were approved by the Institutional Animal Care and Use Committee at the University of Washington.

**Flow cytometry and cell sorting:** Single-cell suspensions from thymus, spleen, and bone marrow were prepared. Red blood cells were removed from splenocytes by water lysis. For flow cytometry, anti-CD16/32 (2.4G2; BD Pharmingen, Franklin Lakes, NJ) was used to block Fc receptors. Cells were stained in Hank’s balanced salt solution containing 1% BSA with fluorochrome-conjugated antibodies (purchased from BD Biosciences or eBioscience, Franklin Lakes, NJ or San Diego, CA) recognizing mouse CD4 (RM4-5), CD8α (53-6.7), CD19 (1D3), CD25 (PC61), CD44 (IM7), CD45R/B220 (RA3-6B2), panTCRβ (H57-597.13), TCRγδ (GL3), Thy1.2 (53-2.1), IgM (II/41), and IgD (11-26c). Flow cytometry data were collected using a FACSCanto (Becton Dickinson, Franklin Lakes, NJ) and analyzed with FlowJo software (Treestar, Ashland, OR).

**Mass Spectrometry:** Free 60S subunits and 80S monosomes from actively translating polysomes were obtained by brief treatment of mouse liver homogenates (prepared as described in Materials and Methods) with RNase A. 8 OD_260_ units of homogenate in 100 µl were incubated on ice for 2 min with 0.4 µg of RNase A to digest inter-ribosomal mRNA, releasing the actively translating 80S ribosomes. Reactions were terminated with addition of 2 M KCl and 20 mg/ml heparin to final concentrations of 460 mM and 0.72 mg/ml, respectively, in a final volume of 139 µl, and loaded onto 4.8-ml 10-30% (w/v) sucrose high-salt (800 mM KCl) gradients as described previously [[1](#_ENREF_1),[2](#_ENREF_2)]. Gradients were centrifuged in a SW50.1 rotor (Beckman Coulter, Brea, CA) at 45,000 rpm (243,000 g) for 2 hr at 4°C. Fractions (0.5 ml) were collected from the top with a Brandel fractionator system and DATAQ DI-148U data recording module as described above. For the 60S subunit samples, fraction 6 from each of three sucrose gradients of a liver lysate from a single KO (*Rpl22^-/-^*) mice were pooled as the “KO60S” sample and fraction 7 the “KO80S” sample, while the same fractions from the three WT (*Rpl22^+/+^*) gradients were pooled to create the “WT60S” and “WT80S” samples. Samples were filtered using 0.8-µm syringe filters (Corning, Corning, NY) and then concentrated and buffer-exchanged with 10 mM Tris buffer using 4-mL, 3-kDa cut-off Amicon Ultra-4 centrifugal filter devices (Millipore, Billerica, MA) operated at 3850 rpm, 4°C for 30-45 min per buffer exchange. A total of 3 buffer exchange iterations were performed, achieving >300x buffer exchange and >10x protein concentration for each sample. All four concentrated samples then underwent standard preparation for mass spectrometry analysis. Briefly, each sample was denatured in 4 M urea, 100 mM ammonium bicarbonate, reduced with 100 mM DTT at 37°C for 30 min, and alkylated with 100 mM iodoacetamide at room temperature for 1 h. The samples were then diluted to 1 M urea using 25 mM ammonium bicarbonate and subjected to protein digestion via overnight incubation at 37°C with 500 ng TPCK-treated trypsin (Sigma-Aldrich, St. Louis, MO). Samples were dried by centrifugal evaporation and cleaned up using C18 UltraMicroSpin columns (The Nest Group, Southborough, MA) per the manufacturer’s instructions. The final eluate for each sample was evaporated and resuspended in 20 µL of 1% acetonitrile, 0.1% formic acid in ultrapure water (Milli-Q, Millipore, Billerica, MA). Multiple Reaction Monitoring Mass Spectrometry (MRM-MS) was used to measure the relative amounts of Rpl22 and Rpl22l1 in the samples. The source for the transition list required by this method was created by a consensus library of previously observed tandem mass spectra using SpectraST [[3](#_ENREF_3),[4](#_ENREF_4)] from the union of two initial spectral libraries: 1) an existing, comprehensive library of MS spectra for the mouse organism, provided by the National Institute of Standards and Technology (NIST) [NIST_mouse_IT_2009-12-10_7AA.splib from http://www.peptideatlas.org/speclib/], and 2) a custom-made library of MS spectra observed in-house from mouse liver lysates and recombinant Rpl22 and Rpl22l1. This library was loaded into Skyline [[5](#_ENREF_5)], which was used to create a transition list for all peptide-fragment pairs (i.e., transitions) that were observed in the consensus spectral library. This final transition list consisted of 4 peptide targets for Rpl22 (5 counting uniquely modified targets) and 3 peptide targets for Rpl22l1, and was limited to the top 8 fragment ions per peptide, creating a total of 64 transitions targeted in the analysis. This transition list was used to program the MRM-MS analysis of the samples described above. Each sample was analyzed in triplicate, with blank runs conducted between each set of three sample runs. All MRM-MS runs were performed on an ABI 4000 QTRAP (Applied Biosystems, Framingham, MA) hybrid quadrupole-ion trap mass spectrometer coupled to a Tempo 2D nano-LC system (Eksigent Technologies, LLC, Dublin, CA) fitted with an LC PACKINGS PepMap 100 reverse-phase C18 column (0.075 x 150 mm). After loading and washing for 5 min with 0.1% formic acid in water (buffer A), peptides were eluted using a linear gradient of 1-30% 0.1% formic acid in acetonitrile (buffer B) over 30 min at a flow rate of 300 nL/min. All runs were performed in positive ion mode with 3000 V of ionization voltage, 30 units of nebulizer gas, a dwell time of 25 ms per transition, and with individual peptide collision energies calculated by Skyline for the ABI platform. Data acquisition was carried out by Analyst software, and the raw data files were loaded into Skyline for analysis.

**Supplemental References**

1. MacKay VL, Li X, Flory MR, Turcott E, Law GL, et al. (2004) Gene expression analyzed by high-resolution state array analysis and quantitative proteomics: response of yeast to mating pheromone. Mol Cell Proteomics 3: 478-489.

2. Martin TE (1973) A simple general method to determine the proportion of active ribosomes in eukaryotic cells. Experimental cell research 80: 496-498.

3. Lam H, Deutsch EW, Eddes JS, Eng JK, King N, et al. (2007) Development and validation of a spectral library searching method for peptide identification from MS/MS. Proteomics 7: 655-667.

4. Lam H, Deutsch EW, Eddes JS, Eng JK, Stein SE, et al. (2008) Building consensus spectral libraries for peptide identification in proteomics. Nature methods 5: 873-875.

5. MacLean B, Tomazela DM, Shulman N, Chambers M, Finney GL, et al. (2010) Skyline: an open source document editor for creating and analyzing targeted proteomics experiments. Bioinformatics 26: 966-968.
